# A prospective, multicenter, comprehensive genomic profile signature study in patients with *EGFR*-mutant advanced non-small cell lung cancer at the first-line treatment failure of osimertinib

**DOI:** 10.1038/s41392-025-02481-8

**Published:** 2025-12-02

**Authors:** Yuankai Shi, Dongqing Lv, Weineng Feng, Shuoyan Liu, Puyuan Xing, Yan Yu, Jun Yin, Xiubao Ren, Junqiang Zhang, Gaohua Han, Yongchang Zhang, Shundong Cang, Jun Chen, Enguo Chen, Lingxin Meng, Yong Zhang

**Affiliations:** 1 Department of Medical Oncology, Beijing Key Laboratory of Key Technologies for Early Clinical Trial Evaluation of Innovative Drugs for Major Diseases, National Cancer Center/National Clinical Research Center for Cancer/Cancer Hospital, Chinese Academy of Medical Sciences & Peking Union Medical College, Beijing, People’s Republic of China; 2 Department of Breath Internal Medicine, Taizhou Hospital of Zhejiang Province, Taizhou, People’s Republic of China; 3 Pulmonary Oncology Department, The First People’s Hospital of Foshan, Foshan, People’s Republic of China; 4 Department of Thoracic Oncology Surgery, Clinical Oncology School of Fujian Medical University, Fujian Cancer Hospital, Fuzhou, People’s Republic of China; 5 Department of Respiratory Medicine, Harbin Medical University Cancer Hospital, Harbin, People’s Republic of China; 6 Pulmonary and Critical Care Medicine, The Third People’s Hospital of Chengdu, Southwest Jiaotong University, Chongqing Medical University, Chengdu, People’s Republic of China; 7 Department of Biotherapy, Tianjin Medical University Cancer Institute and Hospital, Tianjin, People’s Republic of China; 8 Department of Respiratory and Critical Care Medicine, The First Affiliated Hospital of University of Science and Technology of China (Anhui Provincial Hospital), Hefei, People’s Republic of China; 9 Department of Oncology, The People’s Hospital of Taizhou, Taizhou Medical School, Jiangsu and Nantong University, Taizhou, People’s Republic of China; 10 Department of Lung/Gastrointestinal Oncology, Hunan Cancer Hospital, Xiangya School of Medicine, Central South University, Changsha, People’s Republic of China; 11 Department of Oncology, Henan Provincial People’s Hospital, Zhengzhou, People’s Republic of China; 12 Department of Oncology, The Second Hospital of Dalian Medical University, Dalian, The People’s Republic of China; 13 Department of Pulmonary and Critical Care Medicine, Sir Run Run Shaw Hospital, School of Medicine, Zhejiang University, Hangzhou, People’s Republic of China; 14 Department of Oncology, Rizhao People’s Hospital, Rizhao, People’s Republic of China; 15 Department of Pulmonary and Critical Care Medicine, Zhongshan Hospital, Fudan University, Shanghai, People’s Republic of China

**Keywords:** Lung cancer, Cancer genomics

## Abstract

Osimertinib, the first approved third-generation epidermal growth factor receptor (EGFR)-tyrosine kinase inhibitor (TKI), exhibits notable efficacy in *EGFR*-mutant non-small cell lung cancer (NSCLC). This is a prospective, multicenter, comprehensive genomic profile signature (GPS) study in paired tissue and plasma samples from 149 patients with advanced NSCLC harboring *EGFR* exon 19 deletion (Ex19del) or L858R mutation at the first-line treatment failure of osimertinib (NCT05219162). Next-generation sequencing (NGS) was used for comprehensive GPS analysis of paired tissue and plasma samples. Fluorescence in situ hybridization (FISH) and next-generation sequencing (NGS) were used for tissue samples, while droplet digital polymerase chain reaction (ddPCR) and NGS were used for plasma samples to perform a concordance analysis of *MET* amplification. At the first-line treatment failure of osimertinib (study entry), *EGFR* alterations in tissue samples included *EGFR* Ex19del (49.0%, 73/149), *EGFR* L858R mutation (43.0%, 64/149), *EGFR* amplification (32.9%, 49/149), *EGFR* L718Q/V mutation (4.7%, 7/149), and *EGFR* C797S mutation (3.4%, 5/149); bypass signaling activation and downstream pathway activation alterations included *TP53* mutation (69.8%, 104/149) and *MET* amplification (30.9%, 46/149). Among the 136 patients with *EGFR* Ex19del/L858R mutation in tissue samples, 72.1% (98/136), 35.3% (48/136), and 32.4% (44/136) had *TP53* mutations, *EGFR* amplification, and *MET* amplification, respectively. Taking tissue samples as references, the GPS in plasma samples showed high specificity (90.7–100%) for almost all genomic alterations. Compared with FISH (gene copy number [GCN] ≥10), the overall percent agreement of tissue NGS, optimized tissue NGS (GCN ≥ 8.63), plasma NGS, and plasma ddPCR for *MET* amplification were 75.0% (27/36), 100% (36/36), 88.9% (32/36), and 94.4% (34/36), respectively. This study represents the largest-scale, prospective study with paired tissue and plasma samples to enable comprehensive analysis of GPS, providing a novel perspective into coalterations at the first-line treatment failure of osimertinib. A plasma sample serves as a supplement for identifying GPS when a tissue sample is unavailable. Moreover, the integration of FISH, NGS, and ddPCR provided a comprehensive assessment of *MET* amplification.

## Introduction

Osimertinib, the first approved third-generation epidermal growth factor receptor (EGFR)-tyrosine kinase inhibitor (TKI), has demonstrated notable efficacy in patients with advanced non-small cell lung cancer (NSCLC) harboring *EGFR* exon 19 deletion (Ex19del) or L858R mutation, particularly in targeting central nervous system metastases.^1–3^ Given its significant clinical benefits, osimertinib has been widely approved by regulatory authorities, including the U.S. Food and Drug Administration, the European Medicines Agency, and the China National Medical Products Administration, as a first-line treatment for locally advanced or metastatic NSCLC harboring *EGFR* Ex19del/L858R mutation. The PIONEER study revealed that 46.3% (671/1450) of Asian patients and 46.7% (346/741) of mainland Chinese patients with advanced lung adenocarcinoma were *EGFR*-sensitizing mutation positive and suitable for receiving EGFR-TKI therapy.^4,5^

However, most patients ultimately experience treatment failure, and the prognosis is poor for those patients. The ORIENT-31 and HARMONi-A studies demonstrated that the synergistic administration of a programmed cell death protein 1 (PD-1) monoclonal antibody (mAb) and vascular endothelial growth factor (VEGF) mAb, bevacizumab biosimilar IBI305, combined with chemotherapy, or a PD-1/VEGF bispecific antibody, i.e., ivonescimab combined with chemotherapy, significantly improved progression-free survival (PFS) compared with chemotherapy alone or chemotherapy plus placebo in patients upon disease progression after EGFR-TKI therapy.^6,7^ The MARIPOSA-2 study enrolled patients with advanced NSCLC harboring *EGFR* Ex19del/L858R mutation after disease progression on osimertinib treatment and demonstrated that amivantamab plus chemotherapy significantly prolonged both PFS and intracranial PFS compared with chemotherapy alone.^8^ Nevertheless, the inability of these strategies to address disease progression after EGFR-TKI treatment results in a failure to significantly prolong overall survival (OS), highlighting the imperative need for more potent therapeutic approaches.^6,8,9^

Understanding the genomic profile signature (GPS) following progression on EGFR-TKI therapy facilitates the exploration of targeted regimens to improve survival and quality of life in advanced NSCLC patients.^10,11^ Osimertinib overcomes the challenges posed by the *EGFR* T790M mutation, which often arises in patients treated with first- or second-generation EGFR-TKIs.^10,11^ Compared with first-generation EGFR-TKIs, osimertinib significantly improves PFS and OS for patients with *EGFR-*mutant NSCLC in the first-line treatment setting.^1,2^

Investigations into the GPS at the first-line treatment failure of osimertinib are frequently limited by the scarcity of tissue samples at disease progression. Consequently, liquid biopsy has emerged as a viable alternative for identifying and characterizing acquired drug resistance.^12^ Both the AURA3 and FLAURA studies exclusively investigated the GPS at the treatment failure of osimertinib and utilized plasma circulating-tumor DNA (ctDNA), without paired tissue biopsies.^13,14^ However, compared with tissue analysis, ctDNA has limitations in detecting gene mutations, copy number variations (CNVs) and chromosomal rearrangements with reduced accuracy.^12^ The currently available liquid biopsy platforms are incapable of detecting histological transformations when EGFR-TKI treatment fails, thereby limiting their potential as substitutes for tissue biopsy.^15^ Furthermore, most studies of GPS in tissue samples at disease progression during osimertinib treatment tend to have smaller sample sizes. Given the limited accessibility of tumor tissue, exploring the consistency between tissue and plasma GPS data is pivotal for advancing the use of plasma samples as viable alternatives to tissue samples.

Mesenchymal‒epithelial transition (*MET*) amplification and/or overexpression are likely key factors contributing to the first-line treatment failure of osimertinib.^16^ Fluorescence in situ hybridization (FISH) is the gold standard method for detecting *MET* amplification,^17^ whereas immunohistochemistry (IHC) is mainly used to identify *MET* overexpression.^18,19^ The phase 2 INSIGHT2 study identified 33% for FISH 5+ (*MET* gene copy number [GCN] ≥5 or *MET/*centromere of chromosome 7 [CEP7] ≥2),^20^, and the phase 2 SAVANNAH study documented 20% for FISH 10+ (*MET* GCN ≥ 10) and 29% for IHC 90+ (≥90% tumor cells MET IHC 3 + ).^18^ However, high tumor heterogeneity and the scarcity of tissue samples pose significant challenges to the application of FISH.^17^ Next-generation sequencing (NGS) faces challenges in accurately interpreting *MET* amplification due to undefined clinical thresholds and the complexity of distinguishing amplification and polysomy. Droplet digital polymerase chain reaction (ddPCR) is a highly sensitive and specific technique that uses plasma samples for detecting *MET* amplification,^17^ although its diagnostic concordance with tissue FISH requires further confirmation.

Considering the significance of GPS in guiding subsequent treatment, this study focused on exploring tissue and plasma GPS in Chinese patients with advanced NSCLC harboring *EGFR* Ex19del/L858R mutation at the first-line treatment failure of osimertinib (NCT05219162). Additionally, the concordance of NGS and ddPCR with tissue FISH for *MET* amplification detection was investigated in this study, as *MET* amplification is one of the key bypass signaling activation mechanisms contributing to the first-line treatment failure of osimertinib.

## Results

### Patient characteristics at diagnosis and at the first-line treatment failure of osimertinib (study entry)

**Fig. 1:**
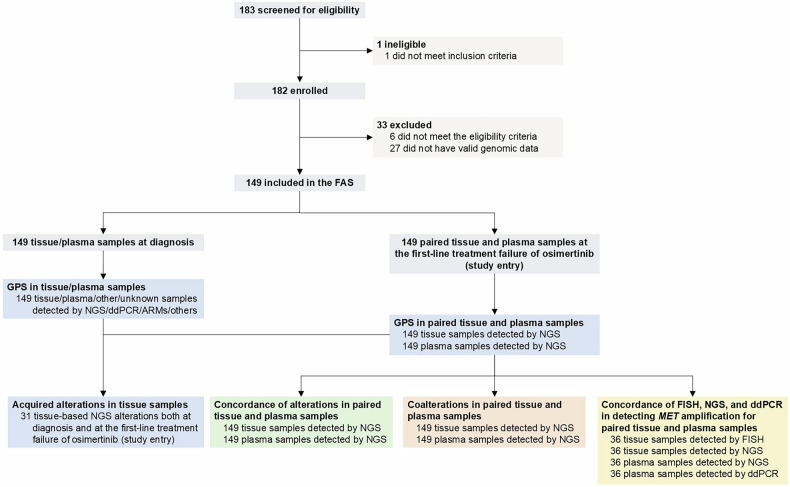
Study flow chart. ARMs, amplification refractory mutation system; ddPCR, droplet digital polymerase chain reaction; FAS, full analysis set; FISH, fluorescence in situ hybridization; GPS, genomic profile signature; MET, mesenchymal‒epithelial transition; NGS, next-generation sequencing

Between February 25, 2022, and April 29, 2024, a total of 182 patients with locally advanced or metastatic NSCLC harboring *EGFR* Ex19del/L858R mutation at the first-line treatment failure of osimertinib were prospectively enrolled. Among these patients, 149 patients with paired tissue and plasma samples were included in the full analysis set (FAS) for final analysis (Fig. 1). Patient characteristics are summarized in Table 1. At diagnosis, 95.3% (142/149) of patients had adenocarcinoma. At the first-line treatment failure of osimertinib (study entry), the median age was 62.0 years (range, 33–86), with 43.6% (65/149) of patients being male and 72.5% (108/149) of patients having never smoked. A total of 98.0% (146/149) of patients had an Eastern Cooperative Oncology Group (ECOG) performance status (PS) of 0–1, and 99.3% (148/149) of patients had stage IV disease.

**Table 1:** Patient characteristics at diagnosis and at the first-line treatment failure of osimertinib (study entry) in the FAS

Characteristic	*N* = 149
Histology type at diagnosis	
Adenocarcinoma	142 (95.3)
Squamous cell carcinoma	2 (1.3)
Adenosquamous carcinoma	2 (1.3)
Mixed adenosquamous-predominant squamous	1 (0.7)
Sarcomatoid carcinoma	1 (0.7)
Other	1 (0.7)
Age at the first-line treatment failure of osimertinib (study entry), years	
Median (range)	62.0 (33–86)
Mean (standard deviation)	62.3 (9.98)
<65 years	84 (56.4)
≥65 years	65 (43.6)
Sex	
Male	65 (43.6)
Female	84 (56.4)
Smoking history at the first-line treatment failure of osimertinib (study entry)	
Current	5 (3.4)
Former	36 (24.2)
Never	108 (72.5)
ECOG PS at the first-line treatment failure of osimertinib (study entry)	
0	29 (19.5)
1	117 (78.5)
≥2	3 (2.0)
Clinical stage at the first-line treatment failure of osimertinib (study entry)	
III	1 (0.7)
IV	148 (99.3)

The data are expressed as n (%) unless otherwise specified

*ECOG* Eastern Cooperative Oncology Group, *FAS* full analysis set, *PS* performance status

### GPS at diagnosis

Among the 149 patients in the FAS, *EGFR* mutations at diagnosis included *EGFR* Ex19del (54.7%, 81/148) and *EGFR* L858R mutation (46.3%, 68/147). For sample type, 77.9% (116/149) of samples were tumor tissue, while for the detection method, 53.0% (79/149) were subjected to NGS (Supplementary Table 1). Among patients with available coalteration data at diagnosis, 28.9% (26/90), 9.5% (11/116), and 2.5% (3/121) of those with tumor protein p53 (*TP53*) mutation, *EGFR* amplification, and *MET* amplification, respectively, were affected (Supplementary Table 2). Among patients harboring concurrent *TP53* mutations, *EGFR* amplifications, and *MET* amplifications, the objective response rates (ORRs) were 46.2% (12/26), 72.7% (8/11), and 66.7% (2/3), respectively (Supplementary Fig. 1 and Supplementary Table 2).

### GPS in tissue samples at the first-line treatment failure of osimertinib (study entry)

**Fig. 2:**
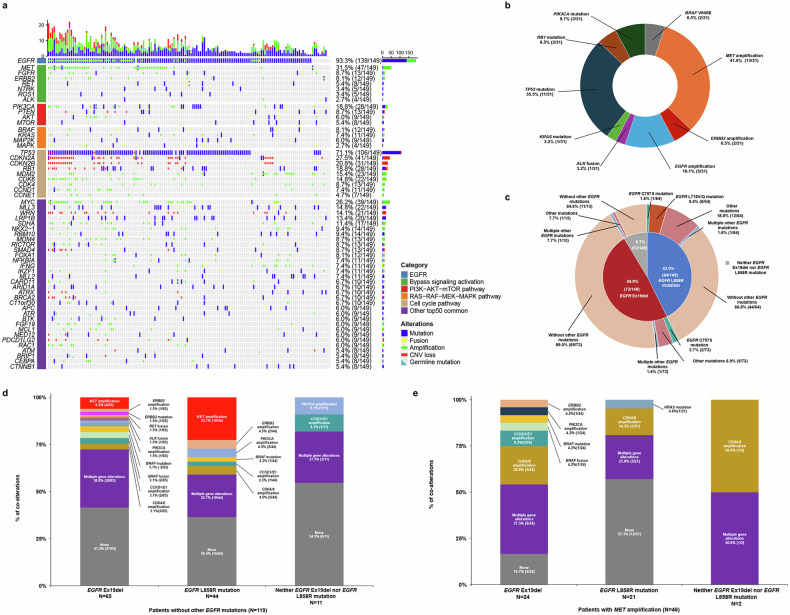
Genomic profile at the first-line treatment failure of osimertinib (study entry) in tissue samples by next-generation sequencing (NGS). **a** Oncoplot of genomic alterations grouped by *EGFR*, bypass signaling activation, downstream pathway activation, and the other top 50 common alterations in the 149 patients at the first-line treatment failure of osimertinib (study entry). **b** Acquired genomic alterations among the 31 patients with tissue-based NGS at the first-line treatment failure of osimertinib (study entry) compared with tissue-based NGS at diagnosis. **c** Coalterations of *EGFR* Ex19del/L858R mutation and other *EGFR* mutations at the first-line treatment failure of osimertinib (study entry). The center circle represents *EGFR* Ex19 del/L858R mutation status among the 149 patients in the FAS. The outer ring represents other *EGFR* mutations. One patient harbored both *EGFR* Ex19del and *EGFR* L858R mutation. Other *EGFR* mutations include *EGFR* mutations, other than *EGFR* Ex19del, *EGFR* L858R, and *EGFR* T790M mutations. **d** Coalterations of bypass signaling activation and downstream pathway activation alterations in the 119 patients without other *EGFR* mutations, grouped by *EGFR* Ex19del, *EGFR* L858R mutation, and neither *EGFR* 19 deletion nor *EGFR* L858R mutation. One patient harbored both *EGFR* Ex19del and *EGFR* L858R mutation. Other *EGFR* mutations include *EGFR* mutations, other than *EGFR* Ex19del, *EGFR* L858R, and *EGFR* T790M mutations. **e** Coalterations in *EGFR*, bypass signaling activation and downstream pathway activation alterations were analyzed in the 46 patients with *MET* amplification, which were grouped into *EGFR* Ex19del, *EGFR* L858R mutation, and neither *EGFR* Ex19del nor *EGFR* L858R mutation. One patient harbored both *EGFR* Ex19del and *EGFR* L858R mutation. Note: Downstream pathways include PI3K − AKT−mTOR pathway, RAS − RAF − MEK − MAPK pathway, and cell cycle pathway. *AKT*, v-akt murine thymoma viral oncogene homolog; *ALK*, anaplastic lymphoma kinase; *APC*, APC regulator of the WNT signaling pathway; *ARID1A*, AT-rich interaction domain 1A; *ATM*, ataxia telangiectasia mutated; *ATR*, ATR serine/threonine kinase; *ATRX*, ATRX chromatin remodeler; *BRAF*, v-raf murine sarcoma viral oncogene homolog B; *BRCA2*, BRCA2 DNA repair associated; *BRIP1*, BRCA1-interacting protein C-terminal helicase 1; *BTK*, Bruton tyrosine kinase; *C11orf30*, chromosome 11 open reading frame 30; *CARD11*, caspase recruitment domain family member 11; *CCND1*, cyclin D1; *CCNE1*, cyclin E1; *CDK4*, cyclin-dependent kinase 4; *CDK6*, cyclin-dependent kinase 6; *CDKN2A*, cyclin-dependent kinase inhibitor 2A; *CDKN2B*, cyclin-dependent kinase inhibitor 2B; *CEBPA*, CCAAT enhancer binding protein alpha; CNV, copy-number variation; *CTNNB1*, catenin beta 1; *EGFR*, epidermal growth factor receptor; *ERBB2*, v-erb-b2 avian erythroblastic leukemia viral oncogene homolog 2; Ex19del, exon 19 deletion; *FGF19*, fibroblast growth factor 19; *FGFR*, fibroblast growth factor receptor; *FOXA1*, forkhead box A1; *IFNG*, interferon gamma; *IKZF1*, IKAROS family zinc finger 1; *KRAS*, Kirsten rat sarcoma viral oncogene homolog; *LRP1B*, LDL receptor related protein 1B; *MAPK*, mitogen-activated protein kinase; *MAP2K*, Mitogen-activated protein kinase; *MCL1*, myeloid cell leukemia 1; *MDM2*, mouse double minute 2, human homolog of; p53-binding protein; *MDM4*, mouse double minute 4, human homolog of; p53-binding protein; *MED12*, MED12, mediator complex subunit 12; *MEK*, alias symbols of MAP2K; *MET*, mesenchymal-epithelial transition; *MLL2*, mixed-lineage leukemia 2; *MLL3*, mixed-lineage leukemia 3; *MTOR*, mechanistic target of rapamycin kinase; *MYC*, v-myc avian myelocytomatosis viral oncogene homolog; *NFKBIA*, NFKB inhibitor alpha; NGS, next-generation sequencing; *NKX2-1*, NK2 homeobox 1; *NTRK*, neurotrophic tyrosine receptor kinase; *PDCD1LG2*, programmed cell death 1 ligand 2; *PIK3CA*, phosphatidylinositol-4,5-bisphosphate 3-kinase catalytic subunit alpha; *PTEN*, phosphatase and tensin homolog; *RAC1*, Rac family small GTPase 1; *RB1*, retinoblastoma 1; *RBM10*, RNA binding motif protein 10; *RET*, rearranged during transfection; *RICTOR*, RPTOR independent companion of MTOR complex 2; *ROS1*, v-ros avian UR2 sarcoma virus oncogene homolog 1; *SDHA*, succinate dehydrogenase complex subunit A; *SMAD4*, SMAD family member 4; *TP53*, tumor protein p53; *WRN*, Werner syndrome RecQ like helicase factor 19; *FGFR*, fibroblast growth factor receptor; *FOXA1*, forkhead box A1; *IFNG*, interferon gamma; *IKZF1*, IKAROS family zinc finger 1; *KRAS*, Kirsten rat sarcoma viral oncogene homolog; *LRP1B*, LDL receptor related protein 1B; *MAPK*, mitogen-activated protein kinase; *MAP2K*, Mitogen-activated protein kinase kinase; *MCL1*, myeloid cell leukemia 1; *MDM2*, mouse double minute 2, human homolog of; p53-binding protein; *MDM4*, mouse double minute 4, human homolog of; p53-binding protein; *MED12*, MED12, mediator complex subunit 12; *MEK*, alias symbols of MAP2K; *MET*, mesenchymal-epithelial transition; *MLL2*, mixed-lineage leukemia 2; *MLL3*, mixed-lineage leukemia 3; *MTOR*, mechanistic target of rapamycin kinase; *MYC*, v-myc avian myelocytomatosis viral oncogene homolog; *NFKBIA*, NFKB inhibitor alpha; NGS, next-generation sequencing; *NKX2-1*, NK2 homeobox 1; *NTRK*, neurotrophic tyrosine receptor kinase; *PDCD1LG2*, programmed cell death 1 ligand 2; *PIK3CA*, phosphatidylinositol-4,5-bisphosphate 3-kinase catalytic subunit alpha; *PTEN*, phosphatase and tensin homolog; *RAC1*, Rac family small GTPase 1; *RB1*, retinoblastoma 1; *RBM10*, RNA binding motif protein 10; *RET*, rearranged during transfection; *RICTOR*, RPTOR independent companion of MTOR complex 2; *ROS1*, v-ros avian UR2 sarcoma virus oncogene homolog 1; *SDHA*, succinate dehydrogenase complex subunit A; *SMAD4*, SMAD family member 4; *TP53*, tumor protein p53; *WRN*, Werner syndrome RecQ like helicase

The most common alterations in tissue samples were *EGFR* (93.3%, 139/149), *TP53* (71.1%, 106/149), and *MET* (31.5%, 47/149) (Fig. 2a). Furthermore, genomic alterations in tissue samples were categorized into *EGFR*, bypass signaling activation, and downstream pathway activation alterations. *EGFR* alterations included *EGFR* Ex19del (49.0%, 73/149), *EGFR* L858R mutation (43.0%, 64/149), *EGFR* amplification (32.9%, 49/149), *EGFR* L718Q/V mutation (4.7%, 7/149), and *EGFR* C797S mutation (3.4%, 5/149). Bypass signaling activation alterations included *MET* amplification (30.9%, 46/149), v-erb-b2 avian erythroblastic leukemia viral oncogene homolog 2 (*ERBB2*) amplification (7.4%, 11/149), and fibroblast growth factor receptor (*FGFR*) amplification (5.4%, 8/149). Downstream pathway activation alterations included *TP53* mutation (69.8%, 104/149), cyclin-dependent kinase inhibitor 2 A (*CDKN2A*) CNV loss (24.2%, 36/149), cyclin-dependent kinase inhibitor 2B (*CDKN2B*) CNV loss (20.8%, 31/149), and phosphatidylinositol-4,5-bisphosphate 3-kinase catalytic subunit alpha (*PIK3CA*) mutation (11.4%, 17/149) (Supplementary Table 3).

With respect to site-specific alterations, the proportion of *MET* amplification was 21.5% (32/149) for copy numbers <5 and 9.4% (14/149) for copy numbers ≥5. Additional amplifications included cyclin-dependent kinase 6 (*CDK6*) (14.8%, 22/149), cyclin-dependent kinase 4 (*CDK4*) (8.7%, 13/149), and *PIK3CA* (8.1%, 12/149). V600E (2.7%, 4/149) was the predominant v-raf murine sarcoma viral oncogene homolog B (*BRAF*) mutation (Supplementary Table 3).

Acquired genomic alterations were assessed among the 31 patients with available tissue-based NGS data from the GPS both at diagnosis and at the first-line treatment failure of osimertinib (study entry). The most common acquired alteration was *MET* amplification (41.9%, 13/31) (Fig. 2b). The ORR of patients with acquired *MET* amplification was 38.5% (5/13) (Supplementary Table 4).

### GPS in plasma samples at the first-line treatment failure of osimertinib (study entry)

**Fig. 3:**
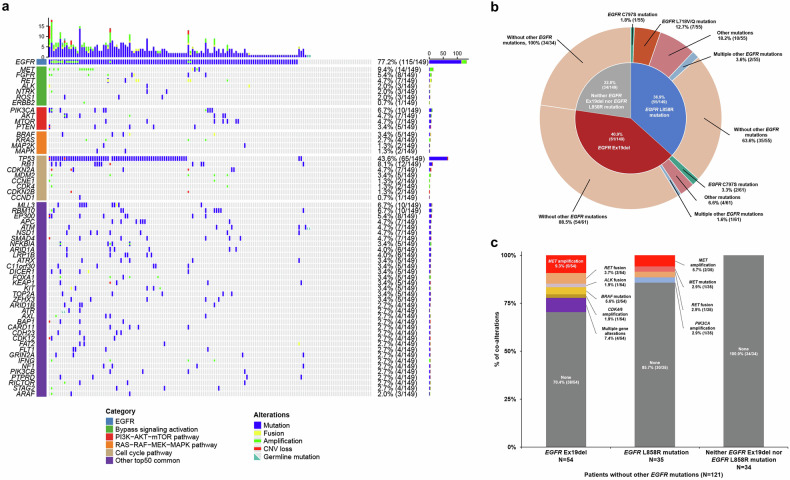
Genomic profile at the first-line treatment failure of osimertinib (study entry) in plasma samples by next-generation sequencing (NGS). **a** Oncoplot of genomic alterations grouped by *EGFR*, bypass signaling activation, downstream pathway activation, and the other top 50 common alterations in the 149 patients at the first-line treatment failure of osimertinib (study entry). **b** Coalterations of *EGFR* Ex19del/L858R mutation and other *EGFR* mutations at the first-line treatment failure of osimertinib (study entry). The center circle represents *EGFR* Ex19del/L858R mutation status among the 149 patients in the FAS. The outer ring represents other *EGFR* mutations. One patient harbored both *EGFR* Ex19del and *EGFR* L858R mutation. Other *EGFR* mutations include *EGFR* mutations, other than *EGFR* Ex19del, *EGFR* L858R, and *EGFR* T790M mutations. **c** *EGFR* alterations in the 121 patients without other *EGFR* mutations, grouped by *EGFR* Ex19del, *EGFR* L858R mutation, and neither E*GFR* 1 Ex19del nor *EGFR* L858R mutation. One patient harbored both *EGFR* 19 deletion and *EGFR* L858R mutation. Note: Downstream pathways include PI3K − AKT−mTOR pathway, RAS − RAF − MEK − MAPK pathway, and cell cycle pathway. *AKT*, v-akt murine thymoma viral oncogene homolog; *ALK*, anaplastic lymphoma kinase; *APC*, APC regulator of the WNT signaling pathway; *ARAF*, v-raf murine sarcoma 3611 viral oncogene homolog 1; *ARID1A*, AT-rich interaction domain 1A; *ARID1B*, AT-rich interaction domain 1B; *ATM*, ataxia telangiectasia mutated; *ATR*, ataxia telangiectasia and Rad3 related; *ATRX*, ATRX chromatin remodeler; *AXL*, AXL receptor tyrosine kinase; BAP1, BRCA1 associated protein 1; *BRAF*, v-raf murine sarcoma viral oncogene homolog B; *C11orf30*, chromosome 11 open reading frame 30; *CARD11*, caspase recruitment domain family member 11; *CCND1*, cyclin D1; *CCNE1*, cyclin E1; *CDH23*, cadherin related 23; *CDK4*, cyclin-dependent kinase 4; *CDK6*, cyclin-dependent kinase 6; *CDK12*, cyclin-dependent kinase 12; *CDKN2A*, cyclin-dependent kinase inhibitor 2 A; *CDKN2B*, cyclin-dependent kinase inhibitor 2B; CNV, copy-number variation; *DICER1*, dicer 1, ribonuclease III; *EGFR*, epidermal growth factor receptor; *EP300*, E1A-binding protein p300; *ERBB2*, Erb-B2 receptor tyrosine kinase 2; *FGF19*, fibroblast growth factor 19; *FAT2*, FAT atypical cadherin 2; *FGFR*, fibroblast growth factor receptor; *FLT1*, fms related receptor tyrosine kinase 1; *FOXA1*, forkhead box A1; *GRIN2A*, glutamate ionotropic receptor NMDA type subunit 2A; *IFNG*, interferon gamma; *KEAP1*, kelch like ECH associated protein 1; *KIT*, v-kit Hardy-Zuckerman 4 feline sarcoma viral oncogene homolog; *KRAS*, Kirsten rat sarcoma viral oncogene homolog; *LRP1B*, LDL receptor related protein 1B; *MAPK*, mitogen-activated protein kinase; *MAP2K*, Mitogen-activated protein kinase; *MDM2*, mouse double minute 2, human homolog of; p53-binding protein; *MET*, mesenchymal-epithelial transition; *MLL3*, mixed-lineage leukemia 3; *MTOR*, mechanistic target of rapamycin kinase; *MYC*, v-myc avian myelocytomatosis viral oncogene homolog; *NF1*, neurofibromin 1; *NFKBIA*, NFKB inhibitor alpha; *NSD1*, nuclear receptor binding SET domain protein 1; NGS, next-generation sequencing; *NTRK*, neurotrophic tyrosine receptor kinase; *PIK3CA*, phosphatidylinositol-4,5-bisphosphate 3-kinase catalytic subunit alpha; *PIK3CB*, phosphatidylinositol-4,5-bisphosphate 3-kinase catalytic subunit beta; *PTEN*, phosphatase and tensin homolog; *PTPRD*, protein tyrosine phosphatase receptor type D; *RB1*, retinoblastoma 1; *RBM10*, RNA binding motif protein 10; *RICTOR*, RPTOR independent companion of MTOR complex 2; *ROS1*, v-ros avian UR2 sarcoma virus oncogene homolog 1; *SMAD4*, SMAD family member 4; *STAG2*, STAG2 cohesin complex component; TOP2A, DNA topoisomerase II alpha; *TP53*, tumor protein P53; *ZFHX3*, zinc finger homeobox 3

*EGFR* (77.2%, 115/149), *TP53* (43.6%, 65/149), and *MET* (9.4%, 14/149) were the most common alterations in plasma samples (Fig. 3a). Genomic alterations in plasma samples were further categorized into *EGFR*, bypass signaling activation, and downstream pathway activation alterations (Fig. 3a). *EGFR* alterations included *EGFR* Ex19del (40.9%, 61/149), *EGFR* L858R mutation (36.9%, 55/149), *EGFR* amplification (10.1%, 15/149), *EGFR* L718Q/V mutation (4.7%, 7/149), and *EGFR* C797S mutation (4.0%, 6/149). Bypass signaling activation alterations included *MET* amplification (7.4%, 11/149), *ERBB2* amplification (0.7%, 1/149), and *FGFR* amplification (2.7%, 4/149) (Supplementary Table 5). Downstream pathway activation alterations included *TP53* mutation (43.6%, 65/149), *RB1* mutation (7.4%, 11/149), and *PIK3CA* mutation (6.0%, 9/149) (Supplementary Table 5).

For site-specific alterations, the proportion of *MET* amplification was 6.0% (9/149) for copy numbers <5 and 1.3% (2/149) for copy numbers ≥5. Additional amplifications included Kirsten rat sarcoma viral oncogene homolog (*KRAS)* (2.7%, 4/149), *CDK4* (1.3%, 2/149), and *PIK3CA* (1.3%, 2/149). The proportion of *PIK3CA* E542K mutation was 2.7% (4/149). V600E (1.3%, 2/149) was the most common *BRAF* mutation (Supplementary Table 5).

### Concordance of alterations in tissue and plasma samples by NGS at the first-line treatment failure of osimertinib (study entry)

**Fig. 4:**
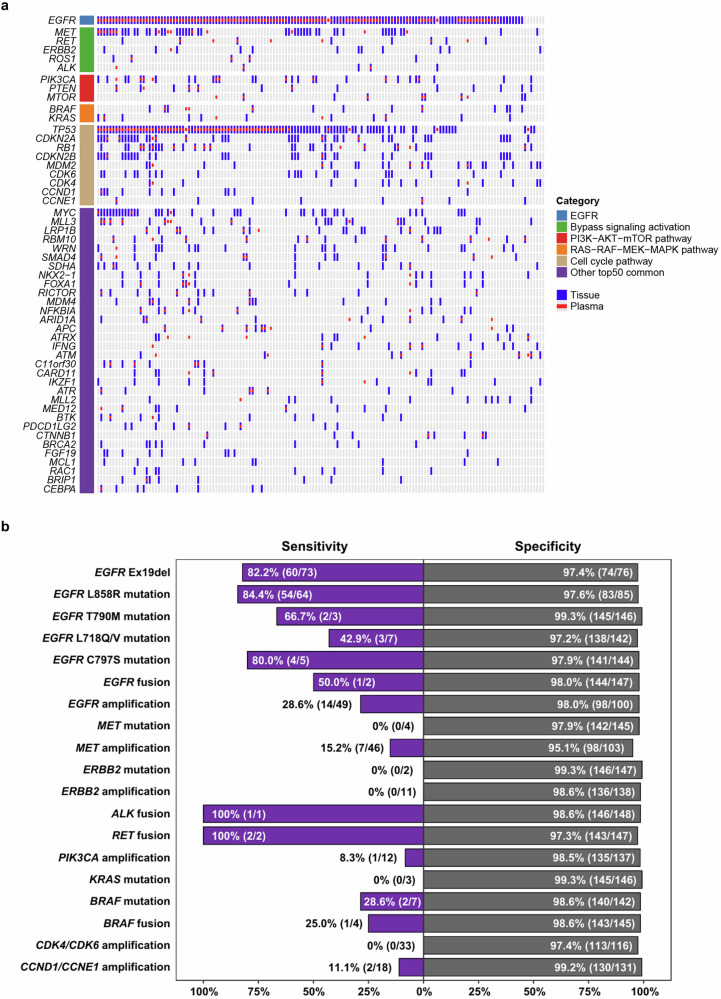
Concordance of NGS detection in tissue and plasma samples at the first-line treatment failure of osimertinib (study entry). **a** Oncoplot plot depicting the concordance of gene expression across tissue and plasma samples. **b** Concordance between tissue and plasma samples for *EGFR*, bypass signaling activation, and downstream pathway activation alterations. Note: Downstream pathways include PI3K − AKT−mTOR pathway, RAS − RAF − MEK − MAPK pathway, and cell cycle pathway .*ALK*, anaplastic lymphoma kinase; *APC*, APC regulator of the WNT signaling pathway; *ARID1A*, AT-rich interaction domain 1A; *ATM*, ataxia telangiectasia mutated; *ATR*, ATR serine/threonine kinase; *ATRX*, ATRX, chromatin remodeler; *BRAF*, v-raf murine sarcoma viral oncogene homolog B; *BRIP1*, BRCA1 interacting protein C-terminal helicase 1; *BTK*, Bruton tyrosine kinase; *C11orf30*, chromosome 11 open reading frame 30; *CARD11*, caspase recruitment domain family member 11; *CCND1*, cyclin D1; *CCNE1*, cyclin E1; *CDK4*, cyclin-dependent kinase 4; *CDKN2A*, cyclin-dependent kinase inhibitor 2A; *CDKN2B*, cyclin-dependent kinase inhibitor 2B; *CEBPA*, CCAAT enhancer binding protein alpha; CNV, copy-number variation; *CTNNB1*, catenin beta 1; *EGFR*, epidermal growth factor receptor; *ERBB2*, v-erb-b2 avian erythroblastic leukemia viral oncogene homolog 2; Ex19del, exon 19 deletion; *FGF19*, fibroblast growth factor 19; *FGFR*, fibroblast growth factor receptor; *FOXA1*, forkhead box A1; *IFNG*, interferon gamma; *IKZF1*, IKAROS family zinc finger 1; *KRAS*, Kirsten rat sarcoma viral oncogene homolog; *LRP1B*, LDL receptor related protein 1B; *MCL1*, myeloid cell leukemia 1; *MDM2*, mouse double minute 2, human homolog of; p53-binding protein; *MDM4*, mouse double minute 4, human homolog of; p53-binding protein; *MED12*, MED12, mediator complex subunit 12; *MEK*, alias symbols of MAP2K; *MET*, mesenchymal-epithelial transition; *MLL2*, mixed-lineage leukemia 2; *MLL3*, mixed-lineage leukemia 3; *MTOR*, mechanistic target of rapamycin kinase; *MYC*, v-myc avian myelocytomatosis viral oncogene homolog; *NFKBIA*, NFKB inhibitor alpha; NGS, next-generation sequencing; *NKX2-1*, NK2 homeobox 1; *PDCD1LG2*, programmed cell death 1 ligand 2; *PIK3CA*, phosphatidylinositol-4,5-bisphosphate 3-kinase catalytic subunit alpha; *PTEN*, phosphatase and tensin homolog; *RB1*, retinoblastoma 1; *RBM10*, RNA binding motif protein 10; *RET*, rearranged during transfection; *RICTOR*, RPTOR independent companion of MTOR complex 2; *ROS1*, v-ros avian UR2 sarcoma virus oncogene homolog 1; *SDHA*, succinate dehydrogenase complex subunit A; *SMAD4*, SMAD family member 4; *TP53*, tumor protein p53; *WRN*, Werner syndrome RecQ like helicase

Using tissue samples detected by NGS as the reference standard, the sensitivity, specificity, and overall percent agreement (OPA) of alterations in plasma were analyzed (Fig. 4 and Supplementary Table 6). Specifically, high specificity (90.7%-100%) was observed in plasma for almost all genomic alterations. The sensitivity and OPA of plasma samples varied: 82.2% (60/73) and 89.9% (134/149) for *EGFR* Ex19del; 84.4% (54/64) and 91.9% (137/149) for *EGFR* L858R mutation; 80.0% (4/5) and 97.3% (145/149) for *EGFR* C797S mutation; 28.6% (14/49) and 75.2% (112/149) for *EGFR* amplification; and 15.2% (7/46) and 70.5% (105/149) for *MET* amplification, respectively (Supplementary Table 6).

### Coalterations in tissue and plasma samples by NGS at the first-line treatment failure of osimertinib (study entry)

With respect to *EGFR* mutations (except for *EGFR* T790M mutation) in tissue samples, 72.5% (108/149) of the patients harbored only *EGFR* Ex19del/L858R mutation, 18.8% (28/149) harbored concurrent *EGFR* Ex19del/L858R mutation and other *EGFR* mutations, 1.3% (2/149) harbored only other *EGFR* mutations, and 7.4% (11/149) harbored no *EGFR* mutations (Supplementary Table 7). Among the 136 patients with *EGFR* Ex19del/L858R mutation in tissue samples, 72.1% (98/136), 35.3% (48/136), and 32.4% (44/136) had *TP53* mutations, *EGFR* amplification, and *MET* amplification, respectively (Supplementary Fig. 2a). The proportion of patients with *EGFR* C797S mutation was 2.7% (2/73) among those with *EGFR* Ex19del and 1.6% (1/64) among those with *EGFR* L858R mutation. *EGFR* L718V/Q mutation was detected in 9.4% (6/64) of patients with *EGFR* L858R mutation and was not detected in patients with *EGFR* Ex19del (Fig. 2c). Among the 108 patients with only *EGFR* Ex19del/L858R mutation in tissue samples, 6.2% (4/65) had concurrent *MET* amplification, and 30.8% (20/65) had other concurrent multiple alterations in patients with *EGFR* Ex19del, whereas 22.7% (10/44) had concurrent *MET* amplification, and 22.7% (10/44) had concurrent multiple alterations in patients with *EGFR* L858R mutation (Fig. 2d). Among the 30 patients with other *EGFR* mutations, two patients had *MET* amplification only, including 1 patient with other mutations and 1 patient with multiple other *EGFR* mutations (Supplementary Table 7). Among the 13 patients without *EGFR* Ex19del/L858R mutation, 46.2% (6/13) had *TP53* mutation (Supplementary Fig. 3a). Among the 11 patients without *EGFR* mutations, none had *MET* amplification, and 54.5% (6/11) had no other alterations (Fig. 2d and Supplementary Table 7).

Among the 46 patients with *MET* amplification in tissue samples, 44 harbored *EGFR* Ex19del/L858R mutation, including *EGFR* Ex19del in 52.2% (24/46) and *EGFR* L858R mutation in 45.7% (21/46) of patients. The proportion of patients with multiple other *EGFR* alterations was 37.5% (9/24) among those with *EGFR* Ex19del and 23.8% (5/21) among those with *EGFR* L858R mutation. The proportion of *CDK4/CDK6* amplification was 20.8% (5/24) in patients with *EGFR* Ex19del and 14.3% (3/21) in those with *EGFR* L858R mutation (Fig. 2e).

With respect to *EGFR* mutations (except for *EGFR* T790M mutation) in plasma samples, 59.1% (88/149) of patients harbored only *EGFR* Ex19del/L858R mutation, 18.1% (27/149) harbored concurrent *EGFR* Ex19del/L858R mutation and other *EGFR* mutations, and 22.8% (34/149) harbored no *EGFR* mutations (Supplementary Table 8). Among the 115 patients with *EGFR* Ex19del/L858R mutation in plasma samples, the proportions of those with *TP53* mutations, *EGFR* amplification, and *MET* amplification were 54.8% (63/115), 13.0% (15/115), and 9.6% (11/115), respectively (Supplementary Fig. 2b). The proportion of patients with *EGFR* C797S mutation was 3.3% (2/61) among those with *EGFR* Ex19del and 1.8% (1/55) among those with *EGFR* L858R mutation. *EGFR* L718V/Q mutation was detected in 12.7% (7/55) of patients with *EGFR* L858R mutation and was not detected in patients with *EGFR* Ex19del (Fig. 3b). Among the 88 patients who harbored only *EGFR* Ex19del/L858R mutation, 9.3% (5/54) had concurrent *MET* amplification, and 7.4% (4/54) had other concurrent multiple alterations in patients with only *EGFR* Ex19del. A total of 5.7% (2/35) of patients with only *EGFR* L858R mutation had concurrent *MET* amplification, and none had concurrent multiple alterations (Fig. 3c and Supplementary Table 8). Among the 27 patients with other *EGFR* mutations, two patients had *MET* amplification only (Supplementary Table 8). Among the 34 patients without *EGFR* Ex19del/L858R mutation, only *TP53* mutations (5.9%, 2/34) and *RB1* mutations (2.9%, 1/34) were observed (Supplementary Fig. 3b). Among the 34 patients without *EGFR* mutations, none had concurrent *MET* amplification or multiple alterations (Fig. 3c and Supplementary Table 8).

### *MET* amplification concordance analysis of NGS or ddPCR compared with FISH at the first-line treatment failure of osimertinib (study entry)

Tissue FISH, tissue NGS, plasma NGS, and plasma ddPCR were concurrently conducted in 36 patients. The rate of positive *MET* amplification was 13.9% (5/36) according to tissue FISH (GCN ≥ 10), 38.9% (14/36) according to tissue NGS, 13.9% (5/36) according to optimized tissue NGS (GCN ≥ 8.63), 8.3% (3/36) according to plasma NGS, and 19.4% (7/36) according to plasma ddPCR (Table 2).

**Table 2:** Proportion of patients with *MET* amplification and interplatform concordance at the first-line treatment failure of osimertinib (study entry) (*N* = 36)

Sample	Methods ^a^	*MET* amplification positive, n (%)	Sensitivity, % (95% CI)	Specificity, % (95% CI)	PPV, % (95% CI)	NPV, % (95% CI)	OPA, % (95% CI)	Kappa, 95% CI
Tissue	FISH ^b^	5 (13.9)	-	-	-	-	-	-
	NGS ^c^	14 (38.9)	100 (47.8, 100)	71.0 (52.0, 85.8)	35.7 (12.8, 64.9)	100 (84.6, 100)	75.0 (57.8, 87.9)	0.404 (0.142, 0.667)
	Optimized NGS ^d^	5 (13.9)	100 (47.8, 100)	100 (88.8, 100)	100 (47.8, 100)	100 (88.8, 100)	100 (90.3, 100)	1.00 (0.673, 1.326)
Plasma	NGS ^c^	3 (8.3)	40.0 (5.3, 85.3)	96.8 (83.3, 99.9)	66.7 (9.4, 99.2)	90.9 (75.7, 98.1)	88.9 (73.9, 96.9)	0.442 (0.128, 0.756)
	ddPCR ^e^	7 (19.4)	100 (47.8, 100)	93.6 (78.6, 99.2)	71.4 (29.0, 96.3)	100 (88.1, 100)	94.4 (81.3, 99.3)	0.801 (0.481, 1.121)

*CI* confidence interval, *ddPCR* droplet digital polymerase chain reaction, *FISH* fluorescence in situ hybridization, *GCN* gene copy number, *MET* mesenchymal‒epithelial transition, *NGS* next-generation sequencing, *NPV* negative predictive value, *OPA* overall percent agreement, *PPV* positive predictive value

a. Tissue FISH (GCN ≥ 10) was used as a reference standard

b. *MET* amplification was detected by FISH in patients with a GCN score ≥10

c. The cutoff value of NGS was set at a copy number ≥2.2

d. The cutoff value of NGS was optimized at a gene copy number ≥8.63

e. The ddPCR threshold was set at a *MET*-to-centromere chromosome 7 ratio of 2 or more (*MET*/CEP7 ≥ 2)

Tissue FISH serving as the reference standard for detecting *MET* amplification, tissue NGS and optimized tissue NGS showed sensitivities of 100% (5/5) and 100% (5/5), specificities of 71.0% (22/31) and 100% (31/31), OPAs of 75.0% (27/36) and 100% (36/36), and kappa values of 0.404 (95% CI 0.142–0.667) and 1.00 (95% CI 0.673–1.326), respectively. The sensitivity, specificity, OPA, and kappa value of plasma NGS were 40.0% (2/5), 96.8% (30/31), 88.9% (32/36), and 0.442 (95% CI 0.128–0.756), respectively. The OPA of plasma ddPCR was 94.4% (34/36), with a sensitivity of 100% (5/5), specificity of 93.6% (29/31), and kappa value of 0.801 (95% CI 0.481–1.121) (Table 2).

Among the 12 patients with available *MET* IHC data, 16.7% (2/12) had *MET* overexpression (IHC 90 + ), with 8.3% (1/12) of patients presenting both *MET* amplification by tissue FISH and *MET* IHC overexpression.

### Pathology transformation at the first-line treatment failure of osimertinib (study entry)

Among the 149 patients with NSCLC in the FAS, 2 (1.3%) patients had pathological transformation from adenocarcinoma to small-cell lung cancer (SCLC), and 2 (1.3%) had transformation from adenocarcinoma to squamous cell carcinoma at the first-line treatment failure of osimertinib (study entry). Among the 2 patients who transformed to SCLC, one patient had *TP53* mutations and *RB1* mutations, and the other patient had *TP53* mutations.

## Discussion

This study, which represents the largest-scale, prospective, multicenter clinical investigation globally in this field, included paired tissue and plasma samples for NGS analysis from 149 patients with *EGFR-*mutant advanced NSCLC at the first-line treatment failure of osimertinib in a real-world setting. An in-depth understanding of the comprehensive GPS at the first-line treatment failure of osimertinib offers the potential for improved therapeutic strategies.

In this study, alterations in *EGFR*, bypass signaling activation, and downstream pathway activation were identified. Among tissue samples, 32.9% (49/149) had *EGFR* amplification, 4.7% (7/149) had *EGFR* L718Q/V mutation, and 3.4% (5/149) had *EGFR* C797S mutation, while the proportions were 10.1% (15/149), 4.7% (7/149), and 4.0% (6/149), respectively, in plasma samples. In previous studies, *EGFR* C797S mutation was identified as the main molecular alteration associated with the first-line treatment failure of osimertinib, occurring in 9.5% (2/21),^21^ 11.1% (3/27),^22^ and 19.6% (9/46)^23^ of patients by tumor tissue detection. Additionally, as a main molecular mechanism of resistance to osimertinib, *EGFR* C797S mutation was acquired in 6.4% (7/109) of patients receiving the first-line treatment of osimertinib by plasma ctDNA detection^14^ and in 1.4% (1/74)^21^ and 15.2% (7/46)^23^ of patients receiving the first-line treatment of osimertinib by tumor tissue detection. *EGFR* amplification has also been identified as a main molecular alteration at the first-line treatment failure of osimertinib, occurring in 3.7% (1/27),^22^ 19.0% (4/21),^21^ and 23.9% (11/46)^23^ of patients by tumor tissue detection. Additionally, as a main molecular mechanism of resistance to osimertinib, *EGFR* amplification was acquired in 4.1% (3/74)^21^ and 10.9% (5/46)^23^ of patients receiving the first-line treatment of osimertinib by tumor tissue detection.

In this study, *TP53* mutation (69.8%, 104/149) and *MET* amplification (30.9%, 46/149) were the most frequent bypass signaling activation and downstream pathway activation alterations in tissue samples, while the proportions were 43.6% (65/149) and 7.4% (11/149) in plasma samples, respectively. In previous studies, *MET* amplification was identified as the main molecular alteration associated with the first-line treatment failure of osimertinib, occurring in 7.4% (2/27),^15^ 9.5% (2/21),^21^ 14.8% (4/27),^22^ and 19.6% (9/46)^23^ of patients by tumor tissue detection. Additionally, as a main molecular mechanism of resistance to osimertinib, *MET* amplification was acquired in 17.9% (14/78)^13^ of patients receiving the second-line treatment of osimertinib by plasma ctDNA detection, in 15.6% (17/109)^14^ of patients receiving the first-line treatment of osimertinib by plasma ctDNA detection, in 7.4% (2/27)^15^ of patients receiving the later-line treatment of osimertinib by tumor tissue detection, and in 7.4% (2/27),^15^ 9.5% (7/74),^21^ and 17.4% (8/46)^23^ of patients receiving the first-line treatment of osimertinib by tumor tissue detection. Cell cycle gene alterations and oncogenic fusions were reported in 16% and 1% of patients who progressed during the first-line treatment of osimertinib, respectively.^14^ A variety of molecular alterations have been identified, suggesting diverse mechanisms underlying treatment failure of osimertinib, underscoring the need for personalized treatment approaches based on specific genomic alterations.

In this study, the best response to the first-line treatment of osimertinib was explored according to genomic alterations at diagnosis. In patients with cooccurring *TP53* mutations, the ORR was 46.2% (12/26) in this study.^21^ In patients with cooccurring *MET* amplification at diagnosis, the ORR was 66.7% (2/3) in this study, whereas it was 61.9% (26/42) for osimertinib 80 mg + savolitinib 300 mg and 66.7% (12/18) for osimertinib 80 mg + savolitinib 600/300 mg in the TATTON study.^18^ Further prospective, randomized controlled studies are needed to determine a more effective treatment strategy for this patient population. This study compared the GPS of patients at diagnosis and at the first-line treatment failure of osimertinib, revealing an acquired *MET* amplification rate of 41.9% (13/31) in tissue samples, which was 15.6% (17/109) in plasma samples in the FLAURA study.^14^ The frequency of acquired *TP53* mutations was 35.5% (11/31) in this study, whereas it was 3% in a prior report.^15^ The discrepancy likely stems from three reasons: incomplete baseline NGS data in routine clinical practice, divergence between diagnostic and treatment failure of osimertinib by NGS assays, and tumor heterogeneity. In this study, acquired alterations at the first-line treatment failure of osimertinib included *TP53*, *RB1*, and *PIK3CA* mutations. Prior studies have consistently linked acquired *TP53* and *RB1* mutations to histologic transformation and poorer clinical outcomes.^10,24^ Additionally, *EGFR-*mutant NSCLC patients who harbored concurrent *PIK3CA* mutations exhibited significantly shorter PFS and OS when treated with EGFR-TKIs.^25^

For patients with limited tissue availability or when repeat biopsies are not feasible, tissue testing could be challenging, and liquid biopsies may be considered alternatives. Therefore, tissue‒plasma concordance of genomic alterations was assessed in this study. Using tissue NGS as the reference, this study demonstrated high specificity of plasma samples for almost all genomic alterations, whereas the sensitivity of plasma samples varied, e.g., 80.0% (4/5) for *EGFR* C797S mutation and 15.2% (7/46) for *MET* amplification. There was a high OPA of 97.3% (145/149) between tissue and plasma NGS for *EGFR* C797S mutation and a moderately high OPA for *EGFR* amplification (75.2%, 112/149) and *MET* amplification (70.5%, 105/149). The AURA3 and FLAURA studies both highlighted the utility of plasma ctDNA analysis in identifying emerging mechanisms underlying treatment failure of osimertinib.^13,14^ Compared with tissue samples, ctDNA offer varying sensitivity for CNVs and chromosomal rearrangements.^15^ This limitation was also observed in the FLAURA study, where it was noted that plasma ctDNA might underestimate amplification events, potentially leading to an expectedly higher frequency of *MET* amplification and *EGFR* amplification in tissue samples.^14^ The high level of concordance in genomic alterations between tissue and plasma samples observed in this study suggests that plasma may serve as an alternative to tissue sample when tissue biopsy is unavailable or insufficient.

In this study, 8.7% (13/149) of patients lost *EGFR* Ex19del*/*L858R mutation in tissue samples at the first-line treatment failure of osimertinib (study entry), reflecting intratumor heterogeneity and clonal replacement.^26^ Additionally, there was a higher *EGFR* mutation negativity rate in plasma samples (22.8%, 34/149) than in tissue samples (8.7%, 13/149), underscoring the value of paired tissue–plasma GPS to capture clonal evolution fully.

*MET* amplification is a predominant resistance mechanism underlying treatment failure of osimertinib, accounting for 17.9% (14/78) of the AURA3 study^13^ and 15.6% (17/109) of the FLAURA study^14^ involving plasma samples. In this study, *MET* amplification was detected in 30.9% (46/149) of tissue samples by NGS at the first-line treatment failure of osimertinib (study entry). Among the 46 patients with *MET* amplification in tissue samples, 95.7% (44/46) presented with *EGFR* Ex19del/L858R mutation, with 24 having a coexisting *EGFR* Ex19del and 21 having *EGFR* L858R mutation. Several early-phase clinical studies have demonstrated that the combination of *EGFR* and *MET* inhibitors is effective for NSCLC patients with *EGFR* mutations and *MET* amplification after EGFR-TKI treatment failure.^27–30^ These findings offer valuable insights to guide the selection of subsequent treatment strategies to improve patient outcomes.

Given the critical role of *MET* amplification in advanced *EGFR-*mutant NSCLC at EGFR-TKI first-line treatment failure and the controversy surrounding detection methods due to small sample sizes and high false-negative rates reported in previous studies,^31^ evaluating concordance among assay methods may inform alternatives to FISH. In this study, *MET* amplification was 13.9% (5/36) by tissue FISH (GCN ≥ 10), 8.3% (3/36) by plasma NGS, and 19.4% (7/36) by plasma ddPCR. Currently, tissue FISH is the gold standard detection method, with *MET* amplification defined as a *MET* GCN ≥ 5 and/or a *MET*/CEP7 ratio ≥2.0.^17,32^ This study used a GCN cutoff value of 10, which is consistent with the SAVANNAH study^18^ and the SACHI study.^33^ In this study, compared with that of tissue FISH, the OPA of plasma ddPCR was 94.4% (34/36), and the kappa value was 0.801, indicating that plasma ddPCR may serve as an alternative when tissue samples are unavailable. Discordance between methods may arise from intratumoral heterogeneity or technical issues (e.g., sampling bias, polysomy vs. focal amplification).^17,34^ Prior studies have suggested that plasma ddPCR may complement tissue FISH in detecting *MET* amplification and guiding MET-targeted therapy. In addition, this study utilized an optimal GCN cutoff of 8.63 for tissue NGS, obtained by comparison with a FISH GCN of 10,^35^ which elevated all the concordance metrics to 100%. When the *MET* GCN cutoff was set at <5 or ≥8 for NGS, the concordance between tissue NGS and tissue FISH improved to 76.5%-100%, whereas a low concordance (34.5%) was observed when the *MET* GCN was ≥5 and <8.^34^ The results of this study corroborated these findings, underscoring the importance of an optimized NGS algorithm for enhancing agreement with FISH and accurately identifying patients with *MET* amplification. Given the small sample size and lack of external validation in this study, the optimal cutoff value for NGS *MET* amplification awaits confirmation in large prospective studies.

In this study, two patients underwent histological transformation from adenocarcinoma to squamous cell carcinoma, and two patients underwent histological transformation from adenocarcinoma to SCLC at the first-line treatment failure of osimertinib. Histologic transformation is recognized at the first-line treatment failure of osimertinib, occurring in 4–7.4% of NSCLC patients with transformation to SCLC and in 5.3–7% of those with squamous cell carcinoma.^15,21,22^ Therefore, tumor tissue biopsy is necessary at the time of failure of osimertinib treatment, since histologic transformation can only be diagnosed on the basis of tumor tissue.

This study had several limitations. The genomic analysis focused on providing insights into subsequent treatment for patients at the first-line treatment failure of osimertinib. The GPS at diagnosis was retrospectively collected, and the association between the GPS at diagnosis and the first-line treatment failure of osimertinib needs further prospective paired studies. The small sample size of patients with *MET* amplification limited the statistical power of the analyses, which were primarily descriptive and may have affected the diagnostic performance metrics of tissue FISH, tissue NGS, plasma NGS, and plasma ddPCR. The efficacy of subsequent treatments based on the GPS at the first-line treatment failure of osimertinib was not monitored in this study.

In conclusion, this study provided prospective paired tumor tissue and plasma samples, allowing for a more comprehensive and accurate analysis of GPS at the first-line treatment failure of osimertinib for *EGFR-*mutant locally advanced or metastatic NSCLC patients. A plasma sample may serve as a supplement for identifying GPS when a tissue sample is unavailable. Moreover, this study offers a novel perspective on coalteration and innovatively combines several methods, including FISH, NGS, and ddPCR, to evaluate *MET* amplification. These findings provide evidence for future studies and clinical practice.

## Materials and methods

### Study design and patients

This was a real-world, multicenter, prospective study (NCT05219162) conducted across 16 hospitals in China. Patients with pathologically confirmed locally advanced or metastatic NSCLC with *EGFR* Ex19del/L858R mutation prior to the first-line treatment of osimertinib were eligible and enrolled. Additional eligibility criteria included evidence of disease progression following the first-line treatment of osimertinib and consent to provide adequate tissue and plasma samples for GPS analysis at disease progression. Patient information, including age, sex, smoking history, histologic type, genetic status, and treatment history, was retrieved from medical records. Tissue-based NGS data of GPS (including *MET* amplification, *ERBB2* amplification, *EGFR* amplification, *ALK* fusion, *ROS1* fusion, *RET* fusion, *NTRK* fusion, *KRAS* mutation, *BRAF* V600E mutation, *TP53* mutation, *RB1* mutation, *PIK3CA* mutation, and *ERBB2* mutation) at diagnosis were collected for analyses of acquired genomic alterations and compared with tissue-based NGS data of GPS at the first-line treatment failure of osimertinib.

The study was approved by the ethics committee of National Cancer Center/Cancer Hospital, Chinese Academy of Medical Sciences & Peking Union Medical College (Approval No. 21/350-3021) and all the institutional ethics committee of each participating hospital. This study was conducted in accordance with the Declaration of Helsinki, the International Council for Harmonization Guideline for Good Clinical Practice, and local regulations. Signed informed consent was obtained from all patients before study entry.

### Sample collection and extraction

Tissue and plasma samples were collected from patients at the first-line treatment failure of osimertinib. Tissue samples with a tumor content of at least 10% were obtained, and 10 mL of peripheral blood was collected and centrifuged to obtain 4 mL of plasma. All the samples were labeled and stored in accordance with standard operating procedures of the accredited central laboratory. Tissue samples were used for the extraction and analysis of deoxyribonucleic acid (DNA), whereas plasma samples were used for the analysis of ctDNA.

### Detection methods

#### NGS

##### DNA extraction

Genomic germline DNA was extracted from peripheral blood lymphocytes (PBLs) with a QIAamp DNA Blood Mini Kit (Qiagen, Hilden, Germany). Genomic DNA was extracted from formalin-fixed, paraffin-embedded (FFPE) tumor tissue samples via the ReliaPrep FFPE gDNA Miniprep System (Promega, Madison, WI, USA). Cell-free DNA (cfDNA) was isolated from 4 mL of plasma via a QIAamp Circulating Nucleic Acid Kit (Qiagen, Hilden, Germany). The size distribution and concentration of cfDNA were assessed via an Agilent 2100 Bioanalyzer (Agilent Technologies, Inc., Santa Clara, CA, USA).

##### Library construction and sequencing

Briefly, genomic DNA from tumor tissues and PBLs was sheared into fragments with a peak of 200 ~ 250 bp with a Covaris S2 ultrasonicator (Covaris, Woburn, MA, USA) before library preparation. The indexed NGS libraries were subsequently constructed from 20–80 ng of cfDNA, 400 ng of paired genomic DNA from PBLs, and 400 ng of paired genomic DNA from tumor tissues via the Hieff NGS Ultima DNA Library Prep Kit for MGI (Yeasen Biotechnology, Shanghai, China). Unique identifiers were labeled on each double-stranded DNA of cfDNA.

The libraries were hybridized to a customized probe set (Integrated DNA Technologies, Inc., Coralville, Iowa, USA) based on a 1.5 Mb region of the 1021-gene panel. After hybridization, the libraries were sequenced on a DNBSEQ-T7RS sequencer (MGI Tech, Shenzhen, China) or a Gene+Seq-2000 sequencing system (Geneplus, Suzhou, China) with 100-bp paired-end reads. On average, 17 Gb of data were generated for cfDNA, 5 Gb of data were generated for tissue DNA, and 1 Gb of data were generated for PBL DNA. Experimental procedures were carried out per the manufacturers’ protocols under restricted quality control and assessment.

The analytical accuracy of NGS exceeded 95%, with the precision reaching 100%. For tissue samples, the limit of detection (LOD) was 3.2 copies for CNVs, 2–5% for single nucleotide variants (SNVs) and insertion-deletions (InDels), and 1–5% for structural variants (SVs). The median sequencing depth was 1300X. For plasma samples, the median sequencing depth was 3000X. The LODs were 3.2 for CNVs, 0.5–1% for SNVs and InDels, and 1% for SVs. The limit of the blank was 0 for both tissue and plasma samples.

#### FISH

*MET* amplification was detected by FISH if sufficient tumor tissue was available. FISH was performed via the Vysis *MET* Spectrum Red FISH Probe Kit (Abbott, Illinois, USA).

#### ddPCR

The ctDNA extracted from plasma sample for examination of *MET* amplification was analyzed via a Bio-Rad Qx200 instrument and a test kit from Shanghai Yuanqi Biopharmaceutical Technology Co., Ltd., China. Approximately 60 μL of the ctDNA was isolated from 2 mL plasma samples via nucleic acid extraction and purification (W005, Shanghai Yuanqi Biopharmaceutical Technology Co., Ltd., China). DG8™ cartridges were gently and steadily placed in a droplet generator to generate droplets, with each well containing 6 μL of ctDNA, and this process was completed within 2 minutes. The ddPCR amplification conditions were as follows: initial denaturation at 95 °C for 10 minutes; 40 cycles of amplification with denaturation at 94 °C for 15 seconds, annealing and extension at 58 °C for 60 seconds; and a final step of reaction termination and incubation at 98 °C for 10 minutes, followed by incubation at 4 °C for 5 minutes. The volume of the reaction system was 40 μL. The CEP7 probe included in the test kit was selected on the basis of published literature, combined with a review of housekeeping gene lists, Bio-Rad guidance documents, and literature related to *ERBB2* amplification. After more than a dozen potential reference genes were evaluated, the CEP7 probe was identified as the optimal reference gene for the kit. The performance of the test kit is summarized as follows: the mixed samples (by mixing *MET*-amplified H1993 cells and *MET*-nonamplified SU-DHL6 cells) were analyzed in 10 independent replicates, demonstrating a coefficient of variation in amplification fold change of <5%. The 20 repeated tests of the LOD references (by mixing *MET*-amplified H1993 cells and *MET*-nonamplified SU-DHL6 cells) showed consistent outcomes, with a *MET* amplification detection rate exceeding 95%. *MET* overexpression was tested by IHC with VENTANA MET (SP44, Roche, Ltd., Basel, Switzerland).

### Definition of genomic alteration categories

Genomic alterations are categorized on the basis of their functional roles in signaling pathways and tumor progression.^25^ These alterations were classified into three main groups: *EGFR*, bypass signaling activation, and downstream pathway activation alterations. *EGFR* alterations included *EGFR* Ex19del/L858R, *EGFR* T790M, and other *EGFR* mutations, which represent *EGFR* mutations except for *EGFR* Ex19del/L858R/T790M mutation, *EGFR* fusion, and *EGFR* amplification. Bypass signaling activation alterations included alterations in *MET*, *ERBB2*, *FGFR*, *RET*, *ALK*, *ROS1*, and *NTRK*. Alterations in downstream pathway activation include alterations in PI3K-AKT-mTOR pathway (e.g., *PIK3CA*, *PTEN*, *AKT*, and *mTOR*), RAS-RAF-MEK-MAPK pathway (e.g., *BRAF*, *KRAS*, *MAPK1/MAPK2/MAPK3*, and *MEK* (*MAP2K1/MAP2K2/MAP2K3/MAP2K4*), and cell cycle pathway (e.g., *CDKN2A/CDKN2B*, *CDK4/CDK6*, CCND*1/CCNE1*, *TP53, RB1*, and *MDM2*).

### Concordance analysis

Concordance analysis of the NGS results between tissue and plasma samples was performed, using tissue NGS as the reference standard. *MET* amplification was tested by FISH and NGS in tissue samples and by ddPCR and NGS in plasma samples. The sensitivity, specificity, positive predictive value (PPV), negative predictive value (NPV), and OPA for *MET* amplification detection were calculated for NGS (tissue and plasma), optimized NGS (tissue), and ddPCR (plasma), with FISH (tissue) used as the reference standard. *MET* amplification positivity was defined as a GCN of ≥10 for tissue FISH according to the SAVANNAH study,^18^ a GCN of ≥8.63 for optimized tissue NGS,^36^ and a GCN of ≥2 for tissue NGS and plasma NGS. The ddPCR threshold was set at a *MET*-to-centromere chromosome 7 ratio of 2 or more (*MET*/CEP7 ≥ 2).

### Statistical analysis

All the statistical analyses were performed via the FAS via SAS software (version 9.4) and R version software (version 4.2.2; R Foundation for Statistical Computing). The FAS included all patients who met the required eligibility criteria and had valid genomic data from both tissue and plasma samples. Descriptive statistics were conducted for all variables as appropriate. Continuous variables were summarized using the mean, standard deviation, median, and range (minimum and maximum). Categorical variables were described as frequencies and percentages. The 95% confidence intervals were calculated whenever appropriate.

## Supplementary information

Supplementary information

## Data Availability

The raw sequence data reported in this paper have been deposited in OMIX, China National Center for Bioinformation/Beijing Institute of Genomics, Chinese Academy of Sciences (OMIX ID: OMIX006547), which is publicly accessible at https://ngdc.cncb.ac.cn/omix.
