# Supplementary material for: A prospective, multicenter, comprehensive genomic profile signature study in patients with EGFR-mutant advanced non-small cell lung cancer at the first-line treatment failure of osimertinib
Source: Signal Transduct Target Ther. 2025 Dec 2;10:393. doi: 10.1038/s41392-025-02481-8 (PMC12669594; doi:10.1038/s41392-025-02481-8)
Supplement: Supplementary file 1 — Supplementary information [file 41392_2025_2481_MOESM1_ESM.docx]

Supplementary Materials for

A prospective, multicenter, comprehensive genomic profile signature (GPS) study in patients with EGFR-mutant advanced non-small cell lung cancer at the first-line treatment failure of osimertinib

Yuankai Shi, Dongqing Lv, Weineng Feng, Shuoyan Liu, Puyuan Xing, Yan Yu, Jun Yin, Xiubao Ren, Junqiang Zhang, Gaohua Han, Yongchang Zhang, Shundong Cang, Jun Chen, Enguo Chen, Lingxin Meng, Yong Zhang

Correspondence to: syuankai@cicams.ac.cn

**This PDF file includes:**

Figures. S1 to S3

Tables S1 to S8


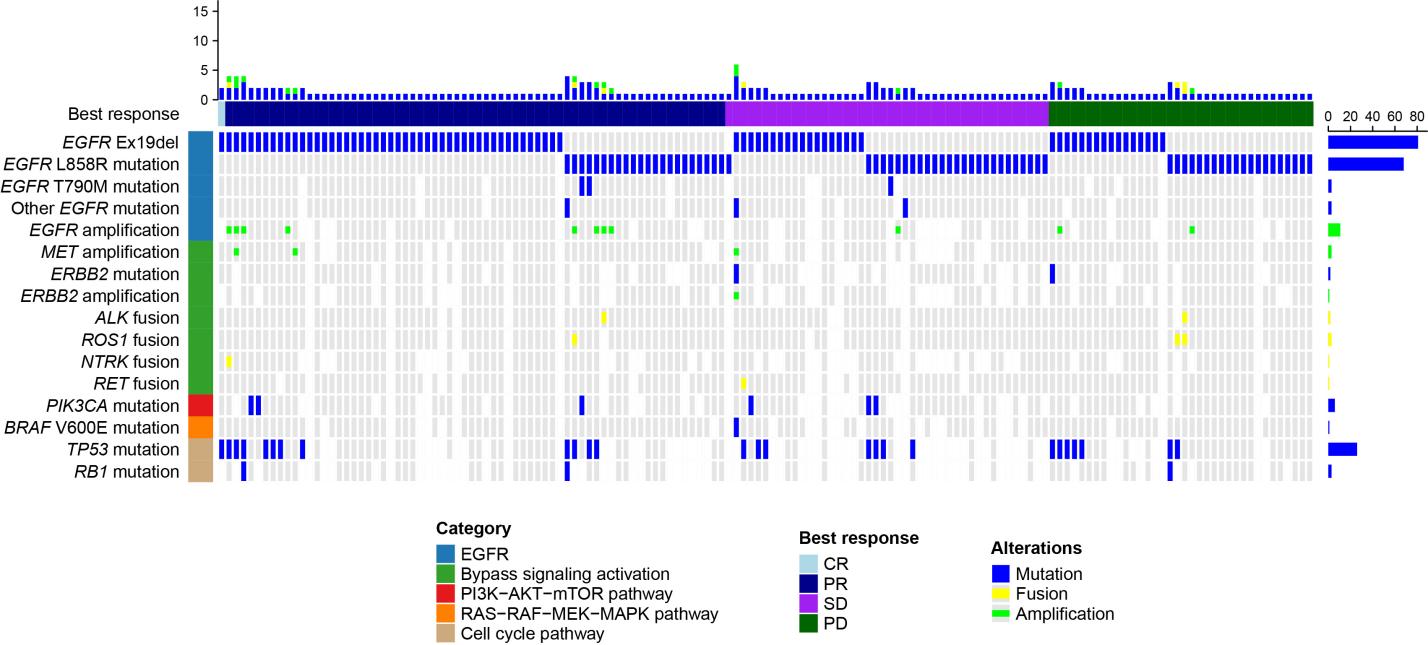


Figure. S1. Oncoplot of GPS at diagnosis and best response to the first-line treatment of osimertinib in the FAS

Note: The downstream pathways include PI3K−AKT−mTOR pathway, RAS−RAF−MEK−MAPK pathway, and cell cycle pathway.

*ALK*, anaplastic lymphoma kinase; *BRAF*, v-raf murine sarcoma viral oncogene homolog B; *EGFR*, epidermal growth factor receptor; *ERBB2*, v-erb-b2 avian erythroblastic leukemia viral oncogene homolog 2; GPS, genomic profile signature; *KRAS*, Kirsten rat sarcoma viral oncogene homolog; *MET*, mesenchymal-epithelial transition; NGS, next-generation sequencing; *NTRK*, neurotrophic tyrosine receptor kinase; *PIK3CA*, phosphatidylinositol-4,5-bisphosphate 3-kinase catalytic subunit alpha; *RB1*, retinoblastoma 1; *RET*, rearranged during transfection; *ROS1*, v-ros avian UR2 sarcoma virus oncogene homolog 1; *TP53*, tumor protein p53


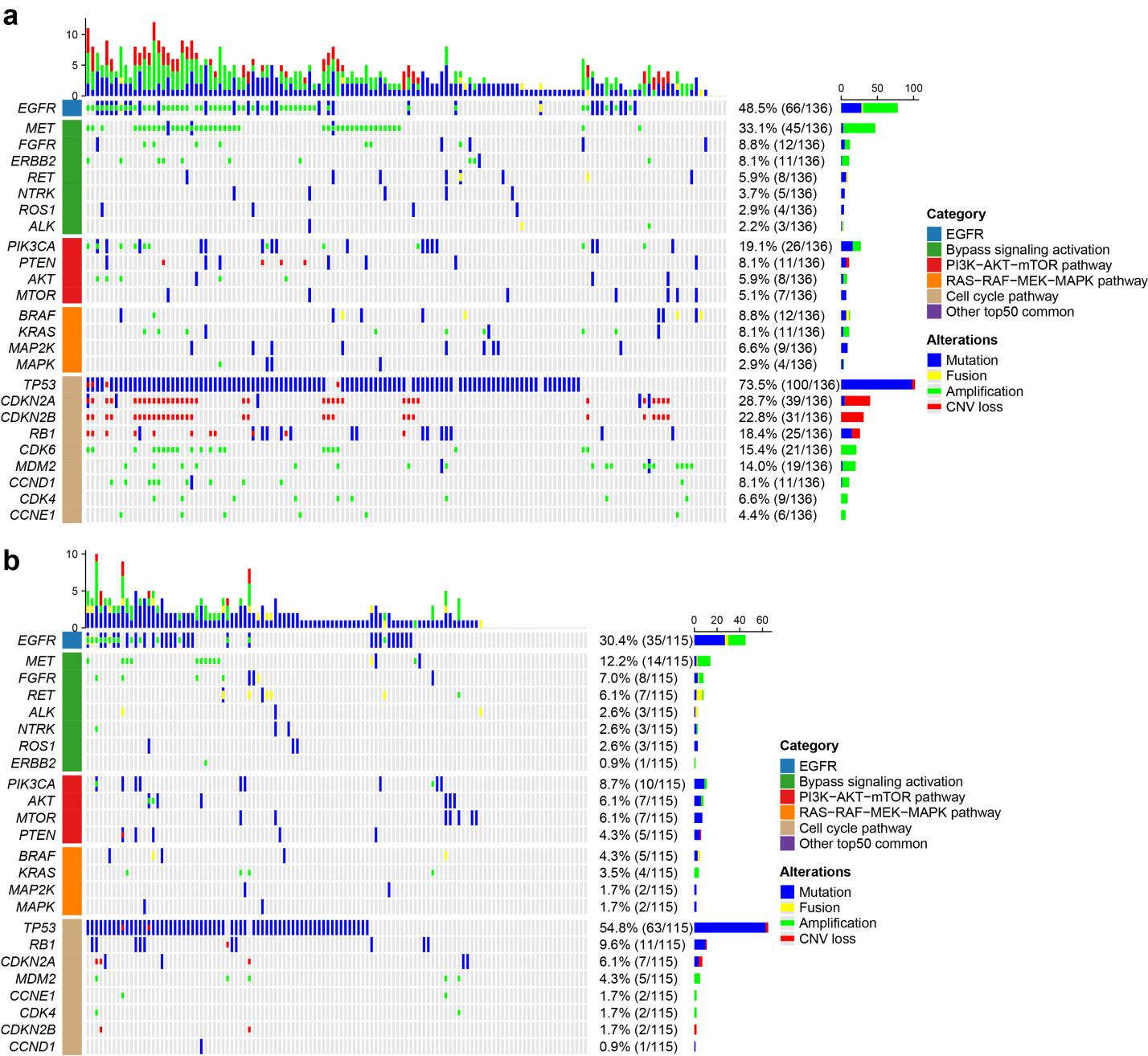


Figure. S2. Oncoplot of GPS for patients with *EGFR* Ex19del/L858R mutation in tissue (n=136) (a) and plasma (n=115) (b) samples at the first-line treatment failure of osimertinib (study entry) by NGS in the FAS

Note: The downstream pathways include PI3K−AKT−mTOR pathway, RAS−RAF−MEK−MAPK pathway, and cell cycle pathway.

*AKT*, v-akt murine thymoma viral oncogene homolog; *ALK*, anaplastic lymphoma kinase; *BRAF*, v-raf murine sarcoma viral oncogene homolog B; *CCND1*, cyclin D1; *CCNE1*, cyclin E1; *CDK4*, cyclin-dependent kinase 4; *CDK6*, cyclin-dependent kinase 6; *CDKN2A*, cyclin-dependent kinase inhibitor 2A; *CDKN2B*, cyclin-dependent kinase inhibitor 2B; CNV, copy-number variation; *CTNNB1*, catenin beta 1; *EGFR*, epidermal growth factor receptor; *ERBB2*, v-erb-b2 avian erythroblastic leukemia viral oncogene homolog 2; Ex19del, exon 19 deletion; *FGF19*, fibroblast growth factor 19; *FGFR*, fibroblast growth factor receptor; GPS, genomic profile signature; *KRAS*, Kirsten rat sarcoma viral oncogene homolog; *MAPK*, mitogen-activated protein kinase; *MAP2K*, Mitogen-activated protein kinase; *MDM2*, mouse double minute 2, human homolog of; p53-binding protein; *MEK*, alias symbols of MAP2K; *MET*, mesenchymal-epithelial transition; *MTOR*, mechanistic target of rapamycin kinase; *MYC*, v-myc avian myelocytomatosis viral oncogene homolog; NGS, next-generation sequencing; *NTRK*, neurotrophic tyrosine receptor kinase; *PIK3CA*, phosphatidylinositol-4,5-bisphosphate 3-kinase catalytic subunit alpha; *PTEN*, phosphatase and tensin homolog; *RB1*, retinoblastoma 1; *RET*, rearranged during transfection; *ROS1*, v-ros avian UR2 sarcoma virus oncogene homolog 1; *TP53*, tumor protein p53.


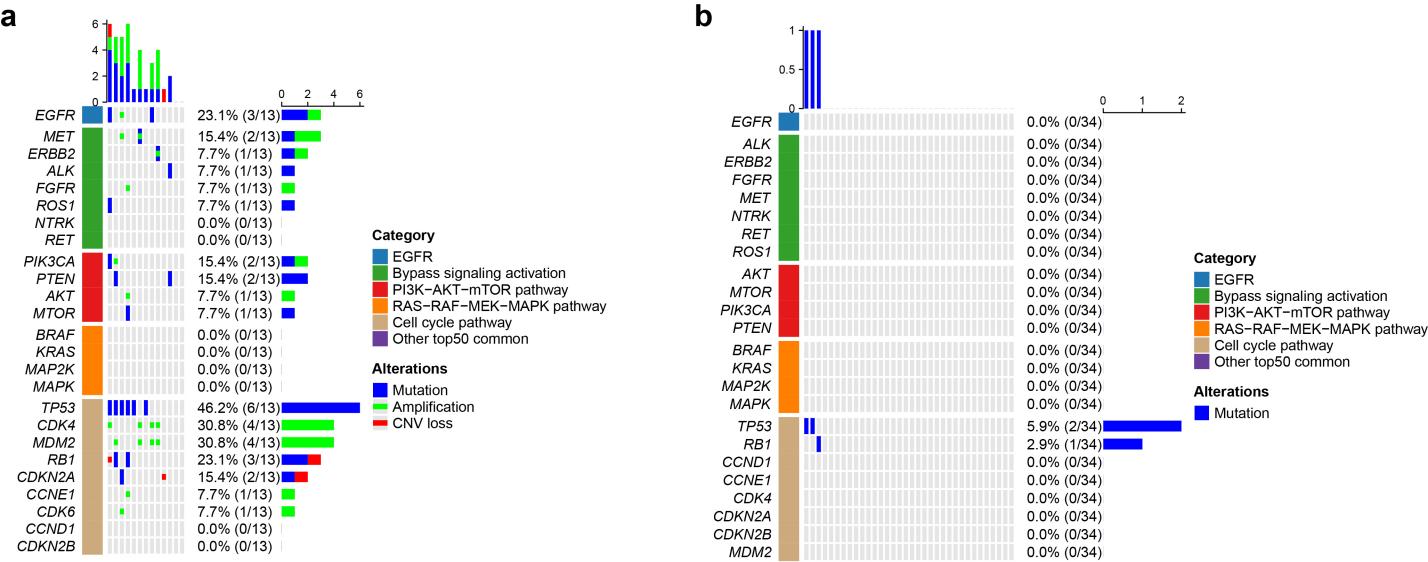


Figure. S3. Oncoplot of GPS for patients without *EGFR* Ex19del/L858R mutation in tissue samples (n=13) (a.) and plasma (n=34) (b.) samples at the first-line treatment failure of osimertinib (study entry) by NGS in the FAS

Note: The downstream pathways include PI3K−AKT−mTOR pathway, RAS−RAF−MEK−MAPK pathway, and cell cycle pathway.

*AKT*, v-akt murine thymoma viral oncogene homolog; *ALK*, anaplastic lymphoma kinase; *BRAF*, v-raf murine sarcoma viral oncogene homolog B; *CCND1*, cyclin D1; *CCNE1*, cyclin E1; *CDK4*, cyclin-dependent kinase 4; *CDK6*, cyclin-dependent kinase 6; *CDKN2A*, cyclin-dependent kinase inhibitor 2A; *CDKN2B*, cyclin-dependent kinase inhibitor 2B; CNV, copy-number variation; *CTNNB1*, catenin beta 1; *EGFR*, epidermal growth factor receptor; *ERBB2*, v-erb-b2 avian erythroblastic leukemia viral oncogene homolog 2; Ex19del, exon 19 deletion; *FGF19*, fibroblast growth factor 19; *FGFR*, fibroblast growth factor receptor; GPS, genomic profile signature; *KRAS*, Kirsten rat sarcoma viral oncogene homolog; *MAPK*, mitogen-activated protein kinase; *MAP2K*, Mitogen-activated protein kinase; *MDM2*, mouse double minute 2, human homolog of; p53-binding protein; *MEK*, alias symbols of MAP2K; *MET*, mesenchymal-epithelial transition; *MTOR*, mechanistic target of rapamycin kinase; *MYC*, v-myc avian myelocytomatosis viral oncogene homolog; NGS, next-generation sequencing; *NTRK*, neurotrophic tyrosine receptor kinase; *PIK3CA*, phosphatidylinositol-4,5-bisphosphate 3-kinase catalytic subunit alpha; *PTEN*, phosphatase and tensin homolog; *RB1*, retinoblastoma 1; *RET*, rearranged during transfection; *ROS1*, v-ros avian UR2 sarcoma virus oncogene homolog 1; *TP53*, tumor protein p53.

Table S1. GPS at diagnosis in the FAS

| **Parameters** | **N=149**  **n (%)** |
| --- | --- |
|  |  |
| **Sample type** |  |
| FFPE tumor | 104 (69.8) |
| Fresh/Frozen tumor | 12 (8.1) |
| Blood | 17 (11.4) |
| Other | 3 (2.0) |
| Unknown | 13 (8.7) |
| **Detection method** |  |
| NGS | 79 (53.0) |
| ddPCR | 15 (10.1) |
| ARMS | 18 (12.1) |
| Other | 37 (24.8) |
| **Alterations ^a^** |  |
| *EGFR* Ex19del | 81/148 (54.7) |
| *EGFR* L858R mutation | 68/147 (46.3) |
| *TP53* mutation | 26/90 (28.9) |
| *EGFR* amplification | 11/116 (9.5) |
| *PIK3CA* mutation | 6/110 (5.5) |
| *RB1* mutation | 3/79 (3.8) |
| *MET* amplification | 3/121(2.5) |
| *EGFR* T790M mutation | 3/136 (2.2) |
| Other *EGFR* mutations ^b^ | 3/134 (2.2) |
| *ROS1* fusion | 3/135 (2.2) |
| *ERBB2* mutation | 2/108 (1.9) |
| *ALK* fusion | 2/136 (1.5) |
| *ERBB2* amplification | 1/102 (1.0) |
| *NTRK* fusion | 1/100 (1.0) |
| *BRAF* V600E mutation | 1/120 (0.8) |
| *RET* fusion | 1/127 (0.8) |

1. Proportions of alterations was calculated among patients with available results of corresponding genes.
2. Other *EGFR* mutations include *EGFR* mutations, other than *EGFR* Ex19del, *EGFR* L858R, and *EGFR* T790M mutations.

*ALK*, anaplastic lymphoma kinase; ARMS, amplification refractory mutation system; *BRAF*, v-raf murine sarcoma viral oncogene homolog B; ddPCR, droplet digital polymerase chain reaction; *EGFR*, epidermal growth factor receptor; *ERBB2*, v-erb-b2 avian erythroblastic leukemia viral oncogene homolog 2; Ex19del, exon 19 deletion; FAS, full analysis set; FFPE, formalin-fixed paraffin-embedded; *MET*, mesenchymal-epithelial transition; NGS, next-generation sequencing; *NTRK*, neurotrophic receptor tyrosine kinase; *PIK3CA,* phosphatidylinositol-4,5-bisphosphate 3-kinase catalytic subunit alpha; *RB1*, retinoblastoma 1; *RET*, rearranged during transfection; *ROS1*, v-ros avian UR2 sarcoma virus oncogene homolog 1; *TP53*, tumor protein p53

Table S2. Objective response to the first-line treatment of osimertinib according to different co-alterations of *EGFR* Ex19del/L858R mutation at diagnosis in the FAS

| **Coalterations** | **Proportion (%)** | **CR (%)** | **PR (%)** | **SD (%)** | **PD (%)** | **ORR (%)** |
| --- | --- | --- | --- | --- | --- | --- |
| *TP53* mutation | 28.9 (26/90) | 3.8 (1/26) | 42.3 (11/26) | 26.9 (7/26) | 26.9 (7/26) | 46.2 (12/26) |
| *EGFR* amplification | 9.5 (11/116) | 0.0 (0/11) | 72.7 (8/11) | 9.1 (1/10) | 18.2 (2/11) | 72.7 (8/11) |
| *PIK3CA* mutation | 5.5 (6/110) | 0.0 (0/6) | 50.0 (3/6) | 50.0 (3/6) | 0.0 (0/6) | 50.0 (3/6) |
| *RB1* mutation | 3.8 (3/79) | 0.0 (0/3) | 66.7 (2/3) | 0.0 (0/3) | 33.3 (1/3) | 66.7 (2/3) |
| *MET* amplification | 2.5 (3/121) | 0.0 (0/3) | 66.7 (2/3) | 33.3 (1/3) | 0.0 (0/3) | 66.7 (2/3) |
| *EGFR* T790M mutation | 2.2 (3/136) | 0.0 (0/3) | 66.7 (2/3) | 33.3 (1/3) | 0.0 (0/3) | 66.7 (2/3) |
| Other *EGFR* mutations ^a^ | 2.2 (3/134) | 0.0 (0/3) | 33.3 (1/3) | 66.7 (2/3) | 0.0 (0/3) | 33.3 (1/3) |
| *ROS1* fusion | 2.2 (3/135) | 0.0 (0/3) | 33.3 (1/3) | 66.7 (2/3) | 0.0 (0/3) | 33.3 (1/3) |
| *ERBB2* mutation | 1.9 (2/108) | 0.0 (0/2) | 0.0 (0/2) | 50.0 (1/2) | 50.5 (1/2) | 0.0 (0/2) |
| *ALK* fusion | 1.5 (2/136) | 0.0 (0/2) | 50.0 (1/2) | 0.0 (0/2) | 0.0 (0/2) | 50.0 (1/2) |
| *ERBB2* amplification | 1.0 (1/102) | 0.0 (0/1) | 0.0 (0/1) | 100 (1/1) | 0.0 (0/1) | 0.0 (0/1) |
| *NTRK* fusion | 1.0 (1/100) | 0.0 (0/1) | 100 (1/1) | 0.0 (0/1) | 0.0 (0/1) | 0.0 (0/1) |
| *BRAF* V600E mutation | 0.8 (1/120) | 0.0 (0/1) | 0.0 (0/1) | 100 (1/1) | 0.0 (0/1) | 0.0 (0/1) |
| *RET* fusion | 0.8 (1/127) | 0.0 (0/1) | 0.0 (0/1) | 100 (1/1) | 0.0 (0/1) | 0.0 (0/1) |

1. Other *EGFR* mutations include EGFR mutations, other than EGFR Ex19del, EGFR L858R, and EGFR T790M mutations.

*ALK*, anaplastic lymphoma kinase; *BRAF*, v-raf murine sarcoma viral oncogene homolog B; CR, complete response; *EGFR*, epidermal growth factor receptor; *ERBB2*, v-erb-b2 avian erythroblastic leukemia viral oncogene homolog 2; Ex19del, exon 19 deletion; FAS, full analysis set; *KRAS*, Kirsten rat sarcoma viral oncogene homolog; *MET*, mesenchymal-epithelial transition; *NTRK*, neurotrophic receptor tyrosine kinase; ORR, objective response rate; PD, progressive disease; *PIK3CA*, phosphatidylinositol-4,5-bisphosphate 3-kinase catalytic subunit alpha; PR, partial response; *RB1*, retinoblastoma 1; *RET*, rearranged during transfection; *ROS1*, v-ros avian UR2 sarcoma virus oncogene homolog 1; SD, stable disease; *TP53*, tumor protein p53

Table S3. Pathway and site-specific alterations in tissue samples at the first-line treatment failure of osimertinib (study entry) by NGS in the FAS

| **Pathway** | | | **Site-specific alterations** | **Positive**  **N=149**  **n (%)** |
| --- | --- | --- | --- | --- |
| *EGFR* alterations | | | *EGFR* | 139 (93.9) |
|  |  |  | *EGFR* mutation | 138 (92.6) |
|  |  |  | *EGFR* Ex19del | 73 (49.0) |
|  |  |  | *EGFR* L858R | 64 (43.0) |
|  |  |  | *EGFR* T790M | 3 (2.0) |
|  |  |  | *EGFR* C797S | 5 (3.4) |
|  |  |  | *EGFR* L718Q/V | 7 (4.7) |
|  |  |  | *EGFR* V689L/M | 3 (2.0) |
|  |  |  | *EGFR* D761N | 2 (1.3) |
|  |  |  | *EGFR* L62R | 2 (1.3) |
|  |  |  | *EGFR* R776H | 2 (1.3) |
|  |  |  | *EGFR* A289T/V | 2 (1.3) |
|  |  |  | *EGFR* D1012N | 1 (0.7) |
|  |  |  | *EGFR* E709A | 1 (0.7) |
|  |  |  | *EGFR* G719A | 1 (0.7) |
|  |  |  | *EGFR* G724S | 1 (0.7) |
|  |  |  | *EGFR* L747V | 1 (0.7) |
|  |  |  | *EGFR* L838V | 1 (0.7) |
|  |  |  | *EGFR* L861Q | 1 (0.7) |
|  |  |  | *EGFR* M952I | 1 (0.7) |
|  |  |  | *EGFR* S306P | 1 (0.7) |
|  |  |  | *EGFR* V308I | 1 (0.7) |
|  |  |  | *EGFR* amplification | 49 (32.9) |
|  |  |  | *EGFR* fusion | 2 (1.3) |
|  |  |  | *EGFR*-LANCL2(intergenic) | 1 (0.7) |
|  |  |  | *EGFR*-ZFAND3 | 1 (0.7) |
| Bypass signaling activation alterations | | | *MET* | 47 (31.5) |
|  |  |  | *MET* mutation | 4 (2.7) |
|  |  |  | *MET* splice site mutation | 1 (0.7) |
|  |  |  | *MET* S406L | 1 (0.7) |
|  |  |  | *MET* S889C | 1 (0.7) |
|  |  |  | *MET* Y321* | 1 (0.7) |
|  |  |  | *MET* amplification | 46 (30.9) |
|  |  |  | *MET* amplification-copy number <5 | 32 (21.5) |
|  |  |  | *MET* amplification-copy number ≥5 | 14 (9.4) |
|  |  |  | *ERBB2* | 12 (8.1) |
|  |  |  | *ERBB2* mutation | 2 (1.3) |
|  |  |  | *ERBB2* E1021K | 1 (0.7) |
|  |  |  | *ERBB2* Y772 A775dup | 1 (0.7) |
|  |  |  | *ERBB2* amplification | 11 (7.4) |
|  |  |  | *FGFR* | 13 (8.7) |
|  |  |  | *FGFR* mutation | 5 (3.4) |
|  |  |  | *FGFR2* Q46* | 1 (0.7) |
|  |  |  | *FGFR2* A266T | 1 (0.7) |
|  |  |  | *FGFR3* S408F | 1 (0.7) |
|  |  |  | *FRGR3* V677I | 1 (0.7) |
|  |  |  | *FGFR4* V262M | 1 (0.7) |
|  |  |  | *FGFR* amplification | 8 (5.4) |
|  |  |  | *FGFR1* amplification | 5 (3.4) |
|  |  |  | *FGFR2* amplification | 1 (0.7) |
|  |  |  | *FGFR3* amplification | 2 (1.3) |
|  |  |  | *FGFR4* amplification | 1 (0.7) |
|  |  |  | *RET* | 8 (5.4) |
|  |  |  | *RET* mutation | 7 (4.7) |
|  |  |  | *RET* D547Y | 1 (0.7) |
|  |  |  | *RET* F735L | 1 (0.7) |
|  |  |  | *RET* R475W | 1 (0.7) |
|  |  |  | *RET* R99W | 1 (0.7) |
|  |  |  | *RET* S401I | 1 (0.7) |
|  |  |  | *RET* S696L | 1 (0.7) |
|  |  |  | *RET* V455F | 1 (0.7) |
|  |  |  | *RET* fusion | 2 (1.3) |
|  |  |  | *RET-CCDC6* | 1 (0.7) |
|  |  |  | *RET-NCOA4* | 1 (0.7) |
|  |  |  | *RET-PARD3* | 1 (0.7) |
|  |  |  | *ALK* | 4 (2.7) |
|  |  |  | *ALK* amplification | 1 (0.7) |
|  |  |  | *ALK* fusion | 1 (0.7) |
|  |  |  | *ALK* -EML4 | 1 (0.7) |
|  |  |  | *ALK* mutation | 2(1.3) |
|  |  |  | *ALK* D1389Y | 1 (0.7) |
|  |  |  | *ALK* E1419K | 1 (0.7) |
|  |  |  | *ROS1* | 5 (3.4) |
|  |  |  | *ROS1* mutation | 5 (3.4) |
|  |  |  | *ROS1* D2213E | 1 (0.7) |
|  |  |  | *ROS1* E1257G | 1 (0.7) |
|  |  |  | *ROS1* F1838V | 1 (0.7) |
|  |  |  | *ROS1* Q1127* | 1 (0.7) |
|  |  |  | *ROS1* W755Sfs*15 | 1 (0.7) |
|  |  |  | *NTRK* | 5 (3.4) |
|  |  |  | *NTRK* mutation | 5 (3.4) |
|  |  |  | *NTRK1* R6Q | 1 (0.7) |
|  |  |  | *NTRK2* Y319C | 1 (0.7) |
|  |  |  | *NTRK3* Q389K | 1 (0.7) |
|  |  |  | *NTRK3* T506A | 1 (0.7) |
|  |  |  | *NTRK3* W13* | 1 (0.7) |
| Downstream pathway activation alterations | PI3K-AKT-mTOR alterations | | *PIK3CA* | 28 (18.8) |
|  |  |  | *PIK3CA* mutation | 17 (11.4) |
|  |  |  | *PIK3CA* E542K | 5 (3.4) |
|  |  |  | *PIK3CA* E545K | 4 (2.7) |
|  |  |  | *PIK3CA* P449T | 2 (1.3) |
|  |  |  | *PIK3CA* D1017H | 1 (0.7) |
|  |  |  | *PIK3CA* E39K | 1 (0.7) |
|  |  |  | *PIK3CA* H1047L | 1 (0.7) |
|  |  |  | *PIK3CA* H1047R | 1 (0.7) |
|  |  |  | *PIK3CA* N345I | 1 (0.7) |
|  |  |  | *PIK3CA* Q546K | 1 (0.7) |
|  |  |  | *PIK3CA* Y1021C | 1 (0.7) |
|  |  |  | *PIK3CA* Y1021N | 1 (0.7) |
|  |  |  | *PIK3CA* amplification | 12 (8.1) |
|  |  |  | *PTEN* | 13 (8.7) |
|  |  |  | *PTEN* mutation | 9 (6.0) |
|  |  |  | *PTEN* splice site mutation | 1 (0.7) |
|  |  |  | *PTEN* C136Y | 1 (0.7) |
|  |  |  | *PTEN* G165* | 1 (0.7) |
|  |  |  | *PTEN* G230V | 1 (0.7) |
|  |  |  | *PTEN* G36V | 1 (0.7) |
|  |  |  | *PTEN* K125E | 1 (0.7) |
|  |  |  | *PTEN* K183* | 1 (0.7) |
|  |  |  | *PTEN* M35V | 1 (0.7) |
|  |  |  | *PTEN* R335* | 1 (0.7) |
|  |  |  | *PTEN* T319*fs*1 | 1 (0.7) |
|  |  |  | *PTEN* Y188Sfs*11 | 1 (0.7) |
|  |  |  | *PTEN* CNV Loss | 4 (2.7) |
|  |  |  | *AKT* | 9 (6.0) |
|  |  |  | *AKT* mutation | 3(2.0) |
|  |  |  | *AKT1* E17K | 1 (0.7) |
|  |  |  | *AKT2* Q429E | 1 (0.7) |
|  |  |  | *AKT3* D246N | 1 (0.7) |
|  |  |  | *AKT* amplification | 6 (4.0) |
|  |  |  | *AKT1* amplification | 2 (1.3) |
|  |  |  | *AKT2* amplification | 3 (2.0) |
|  |  |  | *AKT3* amplification | 1 (0.7) |
|  |  |  | *MTOR* | 8 (5.4) |
|  |  |  | *MTOR* mutation | 8 (5.4) |
|  |  |  | *MTOR* A126V | 1 (0.7) |
|  |  |  | *MTOR* A9V | 1 (0.7) |
|  |  |  | *MTOR* D1736E | 1 (0.7) |
|  |  |  | *MTOR* I1973F | 1 (0.7) |
|  |  |  | *MTOR* K1993N | 1 (0.7) |
|  |  |  | *MTOR* L2230V | 1 (0.7) |
|  |  |  | *MTOR* R1640W | 1 (0.7) |
|  |  |  | *MTOR* S2215Y | 1 (0.7) |
|  | RAS-RAF-MEK-MAPK alterations | | *BRAF* | 12 (8.1) |
|  |  |  | *BRAF* mutation | 7 (4.7) |
|  |  |  | *BRAF* V600E | 4 (2.7) |
|  |  |  | *BRAF* D594N | 1 (0.7) |
|  |  |  | *BRAF* E501K | 1 (0.7) |
|  |  |  | *BRAF* P25S | 1 (0.7) |
|  |  |  | *BRAF* amplification | 1 (0.7) |
|  |  |  | *BRAF* fusion | 4 (2.7) |
|  |  |  | *BRAF-MKRN1* | 1 (0.7) |
|  |  |  | *BRAF-AGK* | 1 (0.7) |
|  |  |  | *BRAF-DPP6* | 1 (0.7) |
|  |  |  | *BRAF-MACF1* | 1 (0.7) |
|  |  |  | *KRAS* | 11 (7.4) |
|  |  |  | *KRAS* mutation | 3 (2.0) |
|  |  |  | *KRAS* A146T | 1 (0.7) |
|  |  |  | *KRAS* G12A | 1 (0.7) |
|  |  |  | *KRAS* Q61H | 1 (0.7) |
|  |  |  | *KRAS* amplification | 8 (5.4) |
|  |  |  | *MAPK* | 4 (2.7) |
|  |  |  | *MAPK* mutation | 3 (2.0) |
|  |  |  | *MAPK1* E322K | 1 (0.7) |
|  |  |  | *MAPK3* E203K | 1 (0.7) |
|  |  |  | *MAPK3* S139R | 1 (0.7) |
|  |  |  | *MAPK* amplification | 1 (0.7) |
|  |  |  | *MAPK1* amplification | 1 (0.7) |
|  |  |  | *MAP2K* | 4 (2.7) |
|  |  |  | *MAP2K* mutation | 9 (6.0) |
|  |  |  | *MAP2K1* E69Q | 1 (0.7) |
|  |  |  | *MAP2K1* D303E | 1 (0.7) |
|  |  |  | *MAP2K1* E203K | 1 (0.7) |
|  |  |  | *MAP2K2* E73K | 1 (0.7) |
|  |  | | *MAP2K4* D289N | 1 (0.7) |
|  |  | | *MAP2K4* A211Qfs*3 | 1 (0.7) |
|  |  | | *MAP2K4* I176T | 1 (0.7) |
|  |  | | *MAP2K4* E352K | 1 (0.7) |
|  |  | | *MAP2K4* N234K | 1 (0.7) |
|  |  | | *MAP2K4* F48L | 1 (0.7) |
|  | Cell cycle gene alterations | | *CDKN2A* | 41 (27.5) |
|  |  |  | *CDKN2A* mutation | 6 (4.0) |
|  |  |  | *CDKN2A* R58* | 2 (1.3) |
|  |  |  | *CDKN2A* Y44Lfs*76 | 1 (0.7) |
|  |  |  | *CDKN2A* Q50L | 1 (0.7) |
|  |  |  | *CDKN2A* E33Q | 1 (0.7) |
|  |  |  | *CDKN2A* R80* | 1 (0.7) |
|  |  |  | *CDKN2A* R22L | 1 (0.7) |
|  |  |  | *CDKN2A* A132Gfs*11 | 1 (0.7) |
|  |  |  | *CDKN2A* CNV Loss | 36 (24.2) |
|  |  |  | *CDKN2B* | 31 (20.8) |
|  |  |  | *CDKN2B* CNV Loss | 31 (20.8) |
|  |  |  | *CDK4* | 13 (8.7) |
|  |  |  | *CDK4* amplification | 13 (8.7) |
|  |  |  | *CDK6* | 22 (14.8) |
|  |  |  | *CDK6* amplification | 22 (14.8) |
|  |  |  | *CCND1* | 11 (7.4) |
|  |  |  | *CCND1* mutation | 1 (0.7) |
|  |  |  | *CCND1* F232L | 1 (0.7) |
|  |  |  | *CCND1* amplification | 10 (6.7) |
|  |  |  | *CCNE1* | 7 (4.7) |
|  |  |  | *CCNE1* amplification | 7 (4.7) |
|  |  | | *TP53* | 106 (71.1) |
|  |  |  | *TP53* mutation | 104 (69.8) |
|  |  |  | *TP53* splice site mutation | 12 (8.1) |
|  |  |  | *TP53* C176F | 3 (2.0) |
|  |  |  | *TP53* E285K | 3 (2.0) |
|  |  |  | *TP53* Y205S | 3 (2.0) |
|  |  |  | *TP53* E180* | 2 (1.3) |
|  |  |  | *TP53* H214R | 2 (1.3) |
|  |  |  | *TP53* R248Q | 2 (1.3) |
|  |  |  | *TP53* R273C | 2 (1.3) |
|  |  |  | *TP53* R280K | 2 (1.3) |
|  |  |  | *TP53* R283P | 2 (1.3) |
|  |  |  | *TP53* Y205C | 2 (1.3) |
|  |  |  | *TP53* A69Gfs*7 | 1 (0.7) |
|  |  |  | *TP53* C238Y | 1 (0.7) |
|  |  |  | *TP53* C242Afs*5 | 1 (0.7) |
|  |  |  | *TP53* C275W | 1 (0.7) |
|  |  |  | *TP53* C277F | 1 (0.7) |
|  |  |  | *TP53* D228*fs*1 | 1 (0.7) |
|  |  |  | *TP53* D281E | 1 (0.7) |
|  |  |  | *TP53* D281H | 1 (0.7) |
|  |  |  | *TP53* D42Y | 1 (0.7) |
|  |  |  | *TP53* E171 V172delinsDF | 1 (0.7) |
|  |  |  | *TP53* E204* | 1 (0.7) |
|  |  |  | *TP53* E258G | 1 (0.7) |
|  |  |  | *TP53* E286Q | 1 (0.7) |
|  |  |  | *TP53* E287* | 1 (0.7) |
|  |  |  | *TP53* E343* | 1 (0.7) |
|  |  |  | *TP53* E51* | 1 (0.7) |
|  |  |  | *TP53* E51Nfs*72 | 1 (0.7) |
|  |  |  | *TP53* E56* | 1 (0.7) |
|  |  |  | *TP53* F113Qfs*5 | 1 (0.7) |
|  |  |  | *TP53* F341S | 1 (0.7) |
|  |  |  | *TP53* G154Rfs*27 | 1 (0.7) |
|  |  |  | *TP53* G245D | 1 (0.7) |
|  |  |  | *TP53* G245S | 1 (0.7) |
|  |  |  | *TP53* G245V | 1 (0.7) |
|  |  |  | *TP53* G262V | 1 (0.7) |
|  |  |  | *TP53* H179Y | 1 (0.7) |
|  |  |  | *TP53* I255F | 1 (0.7) |
|  |  |  | *TP53* K132N | 1 (0.7) |
|  |  |  | *TP53* K164N | 1 (0.7) |
|  |  |  | *TP53* K321Nfs*24 | 1 (0.7) |
|  |  |  | *TP53* K351* | 1 (0.7) |
|  |  |  | *TP53* L111P | 1 (0.7) |
|  |  |  | *TP53* L111R | 1 (0.7) |
|  |  |  | *TP53* L137Q | 1 (0.7) |
|  |  |  | *TP53* L194R | 1 (0.7) |
|  |  |  | *TP53* N239del | 1 (0.7) |
|  |  |  | *TP53* N247 L252del | 1 (0.7) |
|  |  |  | *TP53* P151S | 1 (0.7) |
|  |  |  | *TP53* P223Rfs*23 | 1 (0.7) |
|  |  |  | *TP53* P250L | 1 (0.7) |
|  |  |  | *TP53* P278T | 1 (0.7) |
|  |  |  | *TP53* P27S | 1 (0.7) |
|  |  |  | *TP53* P301Qfs*44 | 1 (0.7) |
|  |  |  | *TP53* Q100* | 1 (0.7) |
|  |  |  | *TP53* Q100K | 1 (0.7) |
|  |  |  | *TP53* Q331* | 1 (0.7) |
|  |  |  | *TP53* R110L | 1 (0.7) |
|  |  |  | *TP53* R156G | 1 (0.7) |
|  |  |  | *TP53* R158H | 1 (0.7) |
|  |  |  | *TP53* R158L | 1 (0.7) |
|  |  |  | *TP53* R175H | 1 (0.7) |
|  |  |  | *TP53* R175L | 1 (0.7) |
|  |  |  | *TP53* R213L | 1 (0.7) |
|  |  |  | *TP53* R249Gfs*96 | 1 (0.7) |
|  |  |  | *TP53* R249W | 1 (0.7) |
|  |  |  | *TP53* R267P | 1 (0.7) |
|  |  |  | *TP53* R273L | 1 (0.7) |
|  |  |  | *TP53* R280G | 1 (0.7) |
|  |  |  | *TP53* R280T | 1 (0.7) |
|  |  |  | *TP53* R282Efs*61 | 1 (0.7) |
|  |  |  | *TP53* R282W | 1 (0.7) |
|  |  |  | *TP53* R342* | 1 (0.7) |
|  |  |  | *TP53* S166* | 1 (0.7) |
|  |  |  | *TP53* S241C | 1 (0.7) |
|  |  |  | *TP53* S315Pfs*20 | 1 (0.7) |
|  |  |  | *TP53* S96Ffs*53 | 1 (0.7) |
|  |  |  | *TP53* T231 H233delinsV | 1 (0.7) |
|  |  |  | *TP53* V147D | 1 (0.7) |
|  |  |  | *TP53* V173L | 1 (0.7) |
|  |  |  | *TP53* V203L | 1 (0.7) |
|  |  |  | *TP53* V216G | 1 (0.7) |
|  |  |  | *TP53* V274L | 1 (0.7) |
|  |  |  | *TP53* Y107 S116del | 1 (0.7) |
|  |  |  | *TP53* Y220C | 1 (0.7) |
|  |  |  | *TP53* CNV Loss | 4 (2.7) |
|  |  |  | *RB1* | 28 (18.8) |
|  |  |  | *RB1* mutation | 17 (11.4) |
|  |  |  | *RB1* splice site mutation | 3 (2.0) |
|  |  |  | *RB1* A14Gfs*13 | 1 (0.7) |
|  |  |  | *RB1* E737V | 1 (0.7) |
|  |  |  | *RB1* E746Gfs*3 | 1 (0.7) |
|  |  |  | *RB1* I124Rfs*6 | 1 (0.7) |
|  |  |  | *RB1* I172Tfs*8 | 1 (0.7) |
|  |  |  | *RB1* L60Ffs*50 | 1 (0.7) |
|  |  |  | *RB1* Q176* | 1 (0.7) |
|  |  |  | *RB1* Q637* | 1 (0.7) |
|  |  |  | *RB1* R320* | 1 (0.7) |
|  |  |  | *RB1* R556* | 1 (0.7) |
|  |  |  | *RB1* S534Vfs*9 | 1 (0.7) |
|  |  |  | *RB1* V654L | 1 (0.7) |
|  |  |  | *RB1* W75* | 1 (0.7) |
|  |  |  | *RB1* W99* | 1 (0.7) |
|  | |  | *RB1* CNV Loss | 12 (8.1) |
|  | |  | *MDM2* | 23 (15.4) |
|  | |  | *MDM2* mutation | 2 (1.3) |
|  | |  | *MDM2* R35K | 1 (0.7) |
|  | |  | *MDM2* R35K | 1 (0.7) |
|  | |  | *MDM2* amplification | 22 (14.8) |

*AKT*, v-akt murine thymoma viral oncogene homolog; *ALK,* anaplastic lymphoma kinase; *BRAF*, v-raf murine sarcoma viral oncogene homolog B; *CDK4*, cyclin-dependent kinase 4; CNV, copy-number variation; *ERBB2*, v-erb-b2 avian erythroblastic leukemia viral oncogene homolog 2; Ex19del, exon 19 deletion; FAS, full analysis set; *KRAS*, Kirsten rat sarcoma viral oncogene homolog; *MET*, mesenchymal-epithelial transition; *MTOR*, mechanistic target of rapamycin kinase; NGS, next-generation sequencing; *NTRK*, neurotrophic tyrosine receptor kinase; *PIK3CA,* phosphatidylinositol-4,5-bisphosphate 3-kinase catalytic subunit alpha; *PTEN*, phosphatase and tensin homolog; *RB1*, retinoblastoma 1; *RET*, rearranged during transfection; *ROS1*, v-ros avian UR2 sarcoma virus oncogene homolog 1; *RTK*, receptor tyrosine kinase; *TP53*, tumor protein p53

Table S4. Proportion of acquired alterations by NGS in tissue samples and their associated best response to the first-line treatment of osimertinib

| **Alteration ^a^** | **Proportion (%)** | **CR (%)** | **PR (%)** | **SD (%)** | **PD (%)** | **ORR (%)** |
| --- | --- | --- | --- | --- | --- | --- |
| *MET* amplification | 41.9 (13/31) | 0.0 (0/13) | 38.5 (5/13) | 30.7 (4/13) | 30.7 (4/13) | 38.5 (5/13) |
| *TP53* mutation | 35.5 (11/31) | 0.0 (0/11) | 18.2 (2/11) | 36.4 (4/11) | 45.5 (5/11) | 18.2 (2/11) |
| *EGFR* amplification | 16.1 (5/31) | 0.0 (0/5) | 0.0 (0/5) | 60.0 (3/5) | 40.0 (2/5) | 0.0 (0/5) |
| *ERBB2* amplification | 16.1 (5/31) | 0.0 (0/5) | 0.0 (0/5) | 60.0 (3/5) | 40.0 (2/5) | 0.0 (0/5) |
| *PIK3CA* mutation | 9.7 (3/31) | 0.0 (0/3) | 0.0 (0/3) | 0.0 (0/3) | 100 (3/3) | 0.0 (0/3) |
| *BRAF* V600E mutation | 6.5 (2/31) | 0.0 (0/2) | 50.0 (1/2) | 0.0 (0/2) | 50.0 (1/2) | 50.0 (1/2) |
| *RB1* mutation | 6.5 (2/31) | 0.0 (0/2) | 0.0 (0/2) | 50.0 (1/2) | 50.0 (1/2) | 0.0 (0/2) |
| *KRAS* mutation | 3.2 (1/31) | 0.0 (0/1) | 0.0 (0/1) | 0.0 (0/1) | 100 (1/1) | 0.0 (0/1) |
| *ALK* fusion | 3.2 (1/31) | 0.0 (0/1) | 0.0 (0/1) | 100 (1/1) | 0.0 (0/1) | 0.0 (0/1) |
| *NTRK* fusion | 0.0 (0/31) | 0.0 (0/0) | 0.0 (0/0) | 0.0 (0/0) | 0.0 (0/0) | 0.0 (0/0) |
| *RET* fusion | 0.0 (0/31) | 0.0 (0/0) | 0.0 (0/0) | 0.0 (0/0) | 0.0 (0/0) | 0.0 (0/0) |
| *ROS1* fusion | 0.0 (0/31) | 0.0 (0/0) | 0.0 (0/0) | 0.0 (0/0) | 0.0 (0/0) | 0.0 (0/0) |
| *EGFR* T790M mutation | 0.0 (0/31) | 0.0 (0/0) | 0.0 (0/0) | 0.0 (0/0) | 0.0 (0/0) | 0.0 (0/0) |
| *ERBB2* mutation | 0.0 (0/31) | 0.0 (0/0) | 0.0 (0/0) | 0.0 (0/0) | 0.0 (0/0) | 0.0 (0/0) |

1. Only patients with valid alteration testing results at diagnosis and disease progression are listed.

*ALK*, anaplastic lymphoma kinase; *BRAF*, v-raf murine sarcoma viral oncogene homolog B; CR, complete response; *EGFR*, epidermal growth factor receptor; *ERBB2*, v-erb-b2 avian erythroblastic leukemia viral oncogene homolog 2; *KRAS*, Kirsten rat sarcoma viral oncogene homolog; *MET*, mesenchymal-epithelial transition; NGS, next-generation sequencing; *NTRK*, neurotrophic receptor tyrosine kinase; ORR, objective response rate; *PIK3CA*, phosphatidylinositol-4,5-bisphosphate 3-kinase catalytic subunit alpha; PD, progressive disease; PR, partial response; *RB1*, retinoblastoma 1; *RET*, rearranged during transfection; *ROS1*, v-ros avian UR2 sarcoma virus oncogene homolog 1; SD, stable disease; *TP53*, tumor protein p53

Table S5. Pathway and site-specific alterations in plasma samples at the first-line treatment failure of osimertinib (study entry) by NGS in the FAS

| **Pathway** | | **Site-specific alterations** | **Positive**  **N=149**  **n (%)** |
| --- | --- | --- | --- |
| *EGFR* alterations | | *EGFR* | 115 (77.2) |
|  |  | *EGFR* mutation | 115 (77.2) |
|  |  | *EGFR* Ex19del | 61 (40.9) |
|  |  | *EGFR* L858R | 55 (36.9) |
|  |  | *EGFR* T790M | 2 (1.3) |
|  |  | *EGFR* C797S | 6 (4.0) |
|  |  | *EGFR* L718Q/V | 7 (4.7) |
|  |  | *EGFR* V689L/M | 3 (2.0) |
|  |  | *EGFR* D761N | 2 (1.3) |
|  |  | *EGFR* G724S | 2 (1.3) |
|  |  | *EGFR* R776H | 2 (1.3) |
|  |  | *EGFR* D1012N | 1 (0.7) |
|  |  | *EGFR* L62R | 1 (0.7) |
|  |  | *EGFR* L747V | 1 (0.7) |
|  |  | *EGFR* L838V | 1 (0.7) |
|  |  | *EGFR* S306P | 1 (0.7) |
|  |  | *EGFR* A289T/V | 1 (0.7) |
|  |  | *EGFR* Q565* | 1 (0.7) |
|  |  | *EGFR* amplification | 15 (10.1) |
|  |  | *EGFR* fusion | 3 (2.0) |
|  |  | *EGFR*-MYCN | 1 (0.7) |
|  |  | *EGFR*-VOPP1 | 1 (0.7) |
|  |  | VOPP1-*EGFR* | 1 (0.7) |
|  |  | *EGFR*-LANCL2 (intergenic) | 1 (0.7) |
| Bypass signaling activation alterations | | *MET* | 14 (9.4) |
|  |  | *MET* mutation | 2 (1.4) |
|  |  | *MET* splice site mutation | 1 (0.7) |
|  |  | *MET* N1113S | 1 (0.7) |
|  |  | *MET* amplification | 11 (7.4) |
|  |  | *MET* amplification-copy number < 5 | 9 (6.0) |
|  |  | *MET* amplification-copy number ≥ 5 | 2 (1.3) |
|  |  | *MET* fusion | 1 (0.7) |
|  |  | *MET*-MET fusion | 1 (0.7) |
|  |  | *ERBB2* | 1 (0.7) |
|  |  | *ERBB2* amplification | 1 (0.7) |
|  |  | *FGFR* | 7 (4.7) |
|  |  | *FGFR* mutation | 3 (2.0) |
|  |  | *FGFR1* N546K | 1 (0.7) |
|  |  | *FGFR2* A266T | 1 (0.7) |
|  |  | *FGFR4* A729V | 1 (0.7) |
|  |  | *FGFR* amplification | 4 (2.7) |
|  |  | *FGFR1* amplification | 1 (0.7) |
|  |  | *FGFR2* amplification | 1 (0.7) |
|  |  | *FGFR3* amplification | 2 (1.3) |
|  |  | *FGFR* fusion | 1 (0.7) |
|  |  | *FGFR2-FAM190B* | 1 (0.7) |
|  |  | *RET* | 7 (4.7) |
|  |  | *RET* mutation | 2 (1.3) |
|  |  | *RET* D547Y | 1 (0.7) |
|  |  | *RET* R475W | 1 (0.7) |
|  |  | *RET* amplification | 1 (0.7) |
|  |  | *RET* fusion | 5 (3.4) |
|  |  | *RET-CCDC6* | 3 (2.0) |
|  |  | *RET-NCOA4* | 1 (0.7) |
|  |  | *RET-PARD3* | 1 (0.7) |
|  |  | *RET-NRG3(intergenic)* | 1 (0.7) |
|  |  | *RET-TRIM33* | 1 (0.7) |
|  |  | *ALK* | 3 (2.0) |
|  |  | *ALK* mutation | 1 (0.7) |
|  |  | *ALK* E1419K | 1 (0.7) |
|  |  | *ALK* fusion | 3 (2.0) |
|  |  | *ALK-EML4* | 2 (1.3) |
|  |  | *ALK-CRIM1(intergenic)* | 1 (0.7) |
|  |  | *ROS1* | 3 (2.0) |
|  |  | *ROS1* mutation | 3 (2.0) |
|  |  | *ROS1* E1257G | 1 (0.7) |
|  |  | *ROS1* Q1127* | 1 (0.7) |
|  |  | *ROS1* W755Sfs*15 | 1 (0.7) |
|  |  | *NTRK* | 3 (2.0) |
|  |  | *NTRK* mutation | 1 (0.7) |
|  |  | *NTRK1* R6Q | 1 (0.7) |
|  |  | *NTRK3* D624G | 1 (0.7) |
|  |  | *NTRK* amplification | 1 (0.7) |
|  |  | *NTRK1* amplification | 1 (0.7) |
| Downstream pathway activation alterations | PI3K-AKT-mTOR alterations | *PIK3CA* | 10 (6.7) |
|  |  | *PIK3CA* mutation | 9 (6.0) |
|  |  | *PIK3CA* E542K | 4 (2.7) |
|  |  | *PIK3CA* E545K | 3 (2.0) |
|  |  | *PIK3CA* E453K | 1 (0.7) |
|  |  | *PIK3CA* E726K | 1 (0.7) |
|  |  | *PIK3CA* N345I | 1 (0.7) |
|  |  | *PIK3CA* P449T | 1 (0.7) |
|  |  | *PIK3CA* Q546K | 1 (0.7) |
|  |  | *PIK3CA* amplification | 2 (1.3) |
|  |  | *PTEN* | 5 (3.4) |
|  |  | *PTEN* mutation | 5 (3.4) |
|  |  | *PTEN* splice site mutation | 2 (1.3) |
|  |  | *PTEN* C136Y | 1 (0.7) |
|  |  | *PTEN* G165* | 1 (0.7) |
|  |  | *PTEN* K183* | 1 (0.7) |
|  |  | *PTEN* M35V | 1 (0.7) |
|  |  | *PTEN* CNV Loss | 1 (0.7) |
|  |  | *AKT* | 7 (4.7) |
|  |  | *AKT* mutation | 6 (4.1) |
|  |  | *AKT1* D323G | 1 (0.7) |
|  |  | *AKT1* E17K | 1 (0.7) |
|  |  | *AKT1* R76H | 1 (0.7) |
|  |  | *AKT3* D246N | 1 (0.7) |
|  |  | *AKT1* E17K | 1 (0.7) |
|  |  | *AKT1* E9dup | 1 (0.7) |
|  |  | *AKT* amplification | 2 (1.3) |
|  |  | *AKT1* CNV Gain | 1 (0.7) |
|  |  | *AKT3* CNV Gain | 1 (0.7) |
|  |  | *MTOR* | 7 (4.7) |
|  |  | *MTOR* mutation | 7 (4.7) |
|  |  | *MTOR* A9V | 1 (0.7) |
|  |  | *MTOR* D1736E | 1 (0.7) |
|  |  | *MTOR* E427del | 1 (0.7) |
|  |  | *MTOR* I1973F | 1 (0.7) |
|  |  | *MTOR* R1386I | 1 (0.7) |
|  |  | *MTOR* R1640W | 1 (0.7) |
|  |  | *MTOR* R553C | 1 (0.7) |
|  |  | *MTOR S2215Y* | 1 (0.7) |
|  | RAS-RAF-MEK-MAPK alterations | *BRAF* | 5 (3.4) |
|  |  | *BRAF* mutation | 3 (2.0) |
|  |  | *BRAF* V600E | 2 (1.3) |
|  |  | *BRAF* P25S | 1 (0.7) |
|  |  | *BRAF* fusion | 2 (1.3) |
|  |  | *BRAF-MKRN1* | 1 (0.7) |
|  |  | *BRAF-AGK* | 1 (0.7) |
|  |  | *BRAF-AGAP1* | 1 (0.7) |
|  |  | *KRAS* | 4 (2.7) |
|  |  | *KRAS* amplification | 4 (2.7) |
|  |  | *MAPK* | 2 (1.3) |
|  |  | *MAPK3* mutation | 3 (2.0) |
|  |  | *MAPK3* E203K | 1 (0.7) |
|  |  | *MAPK3* S139R | 1 (0.7) |
|  |  | *MAP2K* | 2 (1.3) |
|  |  | *MAP2K* mutation | 1 (0.7) |
|  |  | *MAP2K1* E73K | 1 (0.7) |
|  |  | *MAP2K4* N234K  *MAP2K4* F48L | 1 (0.7)  1 (0.7) |
|  | Cell cycle gene alterations | *CDKN2A* | 7 (4.7) |
|  |  | *CDKN2A* mutation | 4 (2.7) |
|  |  | *CDKN2A* Q50L | 1 (0.7) |
|  |  | *CDKN2A* E33Q | 1 (0.7) |
|  |  | *CDKN2A* R58* | 1 (0.7) |
|  |  | *CDKN2A* R22L | 1 (0.7) |
|  |  | *CDKN2A* CNV Loss | 3 (2.0) |
|  |  | *CDKN2B* | 2 (1.3) |
|  |  | *CDKN2B* CNV Loss | 2 (1.3) |
|  |  | *CDK4* | 2 (1.3) |
|  |  | *CDK4* amplification | 2 (1.3) |
|  |  | *CCND1* | 3 (2.0) |
|  |  | *CCND1* mutation | 1 (0.7) |
|  |  | *CCND1* F232L | 1 (0.7) |
|  |  | *CCNE1* | 2 (1.3) |
|  |  | *CCNE1* amplification | 2 (1.3) |
|  |  | *TP53* | 65 (43.6) |
|  |  | *TP53* mutation | 65 (43.6) |
|  |  | *TP53* splice site mutation | 5 (3.4) |
|  |  | *TP53* C176F | 3 (2.0) |
|  |  | *TP53* R248Q | 3 (2.0) |
|  |  | *TP53* E285K | 2 (1.3) |
|  |  | *TP53* R280K | 2 (1.3) |
|  |  | *TP53* Y205S | 2 (1.3) |
|  |  | *TP53* C238Y | 1 (0.7) |
|  |  | *TP53* C242Efs*15 | 1 (0.7) |
|  |  | *TP53* C275W | 1 (0.7) |
|  |  | *TP53* C277F | 1 (0.7) |
|  |  | *TP53* D281E | 1 (0.7) |
|  |  | *TP53* D281H | 1 (0.7) |
|  |  | *TP53* E171 V172delinsDF | 1 (0.7) |
|  |  | *TP53* E204* | 1 (0.7) |
|  |  | *TP53* E258G | 1 (0.7) |
|  |  | *TP53* E286Q | 1 (0.7) |
|  |  | *TP53* E51* | 1 (0.7) |
|  |  | *TP53* E56* | 1 (0.7) |
|  |  | *TP53* F113Qfs*5 | 1 (0.7) |
|  |  | *TP53* F341S | 1 (0.7) |
|  |  | *TP53* G154Rfs*27 | 1 (0.7) |
|  |  | *TP53* G245D | 1 (0.7) |
|  |  | *TP53* G245S | 1 (0.7) |
|  |  | *TP53* G245V | 1 (0.7) |
|  |  | *TP53* H214R | 1 (0.7) |
|  |  | *TP53* I255F | 1 (0.7) |
|  |  | *TP53* K164N | 1 (0.7) |
|  |  | *TP53* L32Sfs*11 | 1 (0.7) |
|  |  | *TP53* N239del | 1 (0.7) |
|  |  | *TP53* P223Rfs*23 | 1 (0.7) |
|  |  | *TP53* P250L | 1 (0.7) |
|  |  | *TP53* P278T | 1 (0.7) |
|  |  | *TP53* Q100* | 1 (0.7) |
|  |  | *TP53* Q331* | 1 (0.7) |
|  |  | *TP53* R110L | 1 (0.7) |
|  |  | *TP53* R156G | 1 (0.7) |
|  |  | *TP53* R158H | 1 (0.7) |
|  |  | *TP53* R158L | 1 (0.7) |
|  |  | *TP53* R175H | 1 (0.7) |
|  |  | *TP53* R196P | 1 (0.7) |
|  |  | *TP53* R249Gfs*96 | 1 (0.7) |
|  |  | *TP53* R249W | 1 (0.7) |
|  |  | *TP53* R267P | 1 (0.7) |
|  |  | *TP53* R273L | 1 (0.7) |
|  |  | *TP53* R280G | 1 (0.7) |
|  |  | *TP53* R280T | 1 (0.7) |
|  |  | *TP53* R282W | 1 (0.7) |
|  |  | *TP53* R283P | 1 (0.7) |
|  |  | *TP53* R342* | 1 (0.7) |
|  |  | *TP53* S106R | 1 (0.7) |
|  |  | *TP53* S241C | 1 (0.7) |
|  |  | *TP53* V173L | 1 (0.7) |
|  |  | *TP53* V203L | 1 (0.7) |
|  |  | *TP53* V274L | 1 (0.7) |
|  |  | *TP53* Y205C | 1 (0.7) |
|  |  | *TP53* Y220C | 1 (0.7) |
|  |  | *TP53* CNV Loss | 2 (1.3) |
|  |  | *RB1* | 12 (8.1) |
|  |  | *RB1* mutation | 11 (7.4) |
|  |  | *RB1* splice site mutation | 2 (1.3) |
|  |  | *RB1* E737V | 1 (0.7) |
|  |  | *RB1* E746Gfs*3 | 1 (0.7) |
|  |  | *RB1* I124Rfs*6 | 1 (0.7) |
|  |  | *RB1* L60Ffs*50 | 1 (0.7) |
|  |  | *RB1 P796S* | 1 (0.7) |
|  |  | *RB1* R320* | 1 (0.7) |
|  |  | *RB1* S534Vfs*9 | 1 (0.7) |
|  |  | *RB1* V654L | 1 (0.7) |
|  |  | *RB1* W75* | 1 (0.7) |
|  |  | *RB1* CNV Loss | 1 (0.7) |
|  |  | *MDM2* | 5 (3.4) |
|  |  | *MDM2* amplification | 5 (3.4) |

*AKT*, v-akt murine thymoma viral oncogene homolog; *ALK,* anaplastic lymphoma kinase; *BRAF*, v-raf murine sarcoma viral oncogene homolog B; *CDK4*, cyclin-dependent kinase 4; CNV, copy-number variation; *ERBB2*, v-erb-b2 avian erythroblastic leukemia viral oncogene homolog 2; Ex19del, exon 19 deletion; FAS, full analysis set; *KRAS*, Kirsten rat sarcoma viral oncogene homolog; *MET*, mesenchymal-epithelial transition; *MTOR*, mechanistic target of rapamycin kinase; NGS, next-generation sequencing; *NTRK1*, neurotrophic tyrosine receptor kinase 1; *PIK3CA,* phosphatidylinositol-4,5-bisphosphate 3-kinase catalytic subunit alpha; *PTEN*, phosphatase and tensin homolog; *RB1*, retinoblastoma 1; *RET*, rearranged during transfection; *ROS1*, v-ros avian UR2 sarcoma virus oncogene homolog 1; *RTK*, receptor tyrosine kinase; *TP53*, tumor protein p53

Table S6. Concordance of top 20 genomic alterations in tissue and plasma samples by NGS at the first-line treatment failure of osimertinib (study entry) in the FAS

| **Alterations ^a^** | **Positive in tissue** | **Positive**  **in plasma** | **Both positive** | **Negative**  **in tissue** | **Negative**  **in plasma** | **Both negative** | **Sensitivity (%)** | **Specificity (%)** | **PPV (%)** | **NPV (%)** | **OPA**  **(%)** |
| --- | --- | --- | --- | --- | --- | --- | --- | --- | --- | --- | --- |
| *EGFR* | 139 | 115 | 113 | 10 | 34 | 8 | 81.3 | 80.0 | 98.3 | 23.5 | 81.2 |
| *EGFR* Ex19del | 73 | 61 | 60 | 76 | 88 | 74 | 82.2 | 97.4 | 98.4 | 84.1 | 89.9 |
| *EGFR* L858R mutation | 64 | 55 | 54 | 85 | 94 | 83 | 84.4 | 97.6 | 98.2 | 88.3 | 91.9 |
| *EGFR* T790M mutation | 3 | 2 | 2 | 146 | 147 | 145 | 66.7 | 99.3 | 100 | 98.6 | 98.7 |
| *EGFR* C797S mutation | 5 | 6 | 4 | 144 | 143 | 141 | 80.0 | 97.9 | 66.7 | 98.6 | 97.3 |
| *EGFR* L718Q/V mutation | 7 | 7 | 3 | 142 | 142 | 138 | 42.9 | 97.2 | 42.9 | 97.2 | 94.6 |
| Other mutations ^b^ | 21 | 16 | 15 | 128 | 133 | 127 | 71.4 | 99.2 | 93.8 | 95.5 | 95.3 |
| *EGFR* amplification | 49 | 15 | 14 | 100 | 134 | 98 | 28.6 | 98.0 | 93.3 | 73.1 | 75.2 |
| *EGFR* fusion | 2 | 3 | 1 | 147 | 146 | 144 | 50.0 | 98.0 | 33.3 | 98.6 | 97.3 |
| *TP53* | 106 | 65 | 61 | 43 | 84 | 39 | 57.5 | 90.7 | 93.8 | 46.4 | 67.1 |
| *MET* | 47 | 14 | 7 | 102 | 135 | 95 | 14.9 | 93.1 | 50.0 | 70.4 | 68.5 |
| *MET* mutation | 4 | 2 | 0 | 145 | 147 | 143 | 0.0 | 96.0 | 0.0 | 98.6 | 96.0 |
| *MET* amplification | 46 | 11 | 7 | 103 | 138 | 98 | 15.2 | 95.1 | 63.6 | 71.0 | 70.5 |
| *CDKN2A* | 41 | 7 | 6 | 108 | 142 | 107 | 14.6 | 99.1 | 85.7 | 75.4 | 75.8 |
| *MYC* | 39 | 3 | 2 | 110 | 146 | 109 | 5.1 | 99.1 | 66.7 | 74.7 | 74.5 |
| *CDKN2B* | 31 | 2 | 2 | 118 | 147 | 118 | 6.5 | 100 | 100 | 80.3 | 80.5 |
| *PIK3CA* | 28 | 10 | 8 | 121 | 139 | 119 | 28.6 | 98.3 | 80.0 | 85.6 | 85.2 |
| *RB1* | 28 | 12 | 10 | 121 | 137 | 119 | 35.7 | 98.3 | 83.3 | 86.9 | 86.6 |
| *MDM2* | 23 | 5 | 4 | 126 | 144 | 125 | 17.4 | 99.2 | 80.0 | 86.8 | 86.6 |
| *CDK6* | 22 | 0 | 0 | 127 | 149 | 127 | 0.0 | 100 | / | 85.2 | 85.2 |
| *MLL3* | 22 | 10 | 7 | 127 | 139 | 124 | 31.8 | 97.6 | 70.0 | 89.2 | 87.9 |
| *WRN* | 21 | 1 | 0 | 128 | 148 | 127 | 0.0 | 99.2 | 0.0 | 85.8 | 85.2 |
| *LRP1B* | 20 | 6 | 5 | 129 | 143 | 128 | 25.0 | 99.2 | 83.3 | 89.5 | 89.3 |
| *SDHA* | 17 | 2 | 2 | 132 | 147 | 132 | 11.8 | 100 | 100 | 89.8 | 89.9 |
| *NKX2-1* | 14 | 3 | 1 | 135 | 146 | 133 | 7.1 | 98.5 | 33.3 | 91.1 | 89.9 |
| *RBM10* | 14 | 10 | 8 | 135 | 139 | 133 | 57.1 | 98.5 | 80.0 | 95.7 | 94.6 |
| *CDK4* | 13 | 2 | 0 | 136 | 147 | 134 | 0.0 | 98.5 | 0.0 | 91.2 | 89.9 |
| *MDM4* | 13 | 3 | 3 | 136 | 146 | 136 | 23.1 | 100 | 100 | 93.2 | 93.3 |
| *SMAD4* | 13 | 5 | 4 | 136 | 144 | 135 | 38.5 | 98.5 | 71.4 | 94.4 | 93.3 |
| *PTEN* | 13 | 7 | 5 | 136 | 142 | 134 | 30.8 | 99.3 | 80.0 | 93.8 | 93.3 |

1. Using tissue samples detected by NGS as the reference standard.
2. Other mutations include *EGFR* V689L/M, *EGFR* D761N, *EGFR* L62R, *EGFR* R776H, *EGFR*A 289T/V, *EGFR* D1012N, *EGFR* E709A, *EGFR* G719A, *EGFR* G724S, *EGFR* L747V, *EGFR* L838V, *EGFR* L861Q, *EGFR* M952I, *EGFR* S306P, *EGFR* V308I, and *EGFR* Q565.

*CDK4,* cyclin-dependent kinase 4; *CDKN2A*, cyclin-dependent kinase inhibitor 2A; *EGFR*, epidermal growth factor receptor; Ex19del, exon 19 deletion; FAS, full analysis set; *LRP1B*, LDL receptor related protein 1B; *MDM2*, mouse double minute 2, human homolog of; p53-binding protein; *MDM4*, mouse double minute 4, human homolog of; p53-binding protein; *MET*, mesenchymal-epithelial transition; *MLL3*, mixed-lineage leukemia 3; *MYC*, v-myc avian myelocytomatosis viral oncogene homolog; NGS, next-generation sequencing; *NKX2-1*, NK2 homeobox 1; NPV, negative predictive value; OPA, overall percent agreement; *PIK3CA,* phosphatidylinositol-4,5-bisphosphate 3-kinase catalytic subunit alpha; PPV, positive predictive value; *PTEN*, phosphatase and tensin homolog; *RB1*, retinoblastoma 1; *RBM10*, RNA-binding motif protein 10; *SDHA*, succinate dehydrogenase complex subunit A; *SMAD4*, SMAD family member 4; *TP53*, tumor protein p53; *WRN*, Werner syndrome RecQ like helicase

Table S7. Coalterations of *EGFR* mutations with bypass signaling activation and downstream pathway activation alterations in tissue samples by NGS at the first-line treatment failure of osimertinib (study entry) in the FAS

|  | *EGFR* Ex19del/L858R mutation only ^a^  N=108 | |  | Other *EGFR* mutations ^b^  N=30 | | | |  | No *EGFR* mutation  N=11  n (%) |
| --- | --- | --- | --- | --- | --- | --- | --- | --- | --- |
|  | *EGFR* Ex19del  N=65  n (%) | *EGFR*  L858R mutation  N=44  n (%) |  | *EGFR* C797S only N=3 n (%) | *EGFR* L718V/Q only N=6 n (%) | Other mutations ^c^ only N=18 n (%) | Multiple other *EGFR* mutations ^b^  N=3 n (%) |  |  |
| **Bypass signaling activation alterations** |  |  |  |  |  |  |  |  |  |
| *MET* amplification only | 4 (6.2) | 10 (22.7) |  | 0 (0.0) | 0 (0.0) | 1 (5.6) | 1 (33.3) |  | 0 (0.0) |
| *MET* mutation only | 0 (0.0) | 0 (0.0) |  | 0 (0.0) | 0 (0.0) | 0 (0.0) | 0 (0.0) |  | 0 (0.0) |
| *ERBB2* amplification only | 1 (1.5) | 2 (4.5) |  | 0 (0.0) | 0 (0.0) | 0 (0.0) | 0 (0.0) |  | 0 (0.0) |
| *ERBB2* mutation only | 1 (1.5) | 0 (0.0) |  | 0 (0.0) | 0 (0.0) | 0 (0.0) | 0 (0.0) |  | 0 (0.0) |
| *RET* fusion only | 1 (1.5) | 0 (0.0) |  | 0 (0.0) | 0 (0.0) | 0 (0.0) | 0 (0.0) |  | 0 (0.0) |
| *ALK* fusion only | 1 (1.5) | 0 (0.0) |  | 0 (0.0) | 0 (0.0) | 0 (0.0) | 0 (0.0) |  | 0 (0.0) |
| **Downstream pathway activation alterations** |  |  |  |  |  |  |  |  |  |
| *PIK3CA* amplification only | 1 (1.5) | 2 4.5) |  | 0 (0.0) | 0 (0.0) | 1 (5.6) | 0 (0.0) |  | 1 (9.1) |
| *KRAS* mutation only | 1 (1.5) | 0 (0.0) |  | 0 (0.0) | 0 (0.0) | 0 (0.0) | 0 (0.0) |  | 0 (0.0) |
| *BRAF* mutation only | 2 (3.1) | 1 (2.3) |  | 0 (0.0) | 0 (0.0) | 0 (0.0) | 0 (0.0) |  | 0 (0.0) |
| *BRAF* fusion only | 2 (3.1) | 0 (0.0) |  | 0 (0.0) | 0 (0.0) | 0 (0.0) | 0 (0.0) |  | 0 (0.0) |
| *CCND1/CCNE1* amplification only | 2 (3.1) | 1 (2.3) |  | 1 (33.3) | 0 (0.0) | 0 (0.0) | 1 (33.3) |  | 1 (9.1) |
| *CDK4/CDK 6* amplification only | 2 (3.1) | 2 (4.5) |  | 0 (0.0) | 0 (0.0) | 2 (11.1) | 1 (33.3) |  | 0 (0.0) |
| **Multiple of the above** | 20 (30.8) | 10 (22.7) |  | 0 (0.0) | 1 (16.7) | 4 (22.2) | 0 (0.0) |  | 3 (27.3) |
| **None of the above** | 27 (41.5) | 16 (36.4) |  | 2 (66.7) | 5 (83.3) | 10 (55.6) | 0 (0.0) |  | 6 (54.5) |

1. One patient harbored both *EGFR* Ex19del and *EGFR* L858R mutation.
2. Other *EGFR* mutations include *EGFR* mutations, other than *EGFR* Ex19del, *EGFR* L858R, and *EGFR* T790M mutations.
3. Other mutations include *EGFR* V689L/M, *EGFR* D761N, *EGFR* L62R, *EGFR* R776H, *EGFR* A289T/V, *EGFR* D1012N, *EGFR* E709A, *EGFR* G719A, *EGFR* G724S, *EGFR* L747V, *EGFR* L838V, *EGFR* L861Q, *EGFR* M952I, *EGFR* S306P, *EGFR* V308I, and *EGFR* Q565.

*ALK*, anaplastic lymphoma kinase; *BRAF*, v-raf murine sarcoma viral oncogene homolog B; *CCND1*, cyclin D1; *CCNE1*, cyclin E1; *CDK4*, cyclin-dependent kinase 4; *CDK6*, cyclin-dependent kinase 6; *EGFR*, epidermal growth factor receptor; *ERBB2*, v-erb-b2 avian erythroblastic leukemia viral oncogene homolog 2; Ex19del, exon 19 deletion; FAS, full analysis set; *KRAS*, Kirsten rat sarcoma viral oncogene homolog; *MET*, mesenchymal-epithelial transition; *PIK3CA*, phosphatidylinositol-4,5-bisphosphate 3-kinase catalytic subunit alpha; *RET*, rearranged during transfection.

Table S8. Coalterations of *EGFR* mutations with bypass signaling activation and downstream pathway activation alterations in plasma samples by NGS at the first-line treatment failure of osimertinib (study entry) in the FAS

|  | *EGFR* Ex19del/L858R mutation only ^a^  N=88 | |  | Other *EGFR* mutations ^b^  N=27 | | | |  | No *EGFR* mutation  N=34  n (%) |
| --- | --- | --- | --- | --- | --- | --- | --- | --- | --- |
|  | *EGFR* Ex19del  N=54  n (%) | *EGFR* L858R mutation  N=35  n (%) |  | *EGFR* C797S only N=3 n (%) | *EGFR* L718V/Q only N=7 n (%) | Other mutations ^c^ only N=14  n (%) | Multiple other *EGFR* mutations ^b^ N=3 n (%) |  |  |
| **Bypass signaling activation alterations** |  |  |  |  |  |  |  |  |  |
| *MET* amplification only | 5 (9.3) | 2 (5.7) |  | 0 (0.0) | 0 (0.0) | 2 (14.3) | 0 (0.0) |  | 0 (0.0) |
| *MET* mutation only | 0 (0.0) | 1 (2.9) |  | 0 (0.0) | 0 (0.0) | 1 (7.1) | 0 (0.0) |  | 0 (0.0) |
| *ERBB2* amplification only | 0 (0.0) | 0 (0.0) |  | 0 (0.0) | 0 (0.0) | 0 (0.0) | 0 (0.0) |  | 0 (0.0) |
| *ERBB2* mutation only | 0 (0.0) | 0 (0.0) |  | 0 (0.0) | 0 (0.0) | 0 (0.0) | 0 (0.0) |  | 0 (0.0) |
| *RET* fusion only | 3 (5.6) | 1 (2.9) |  | 0 (0.0) | 0 (0.0) | 1 (7.1) | 0 (0.0) |  | 0 (0.0) |
| *ALK* fusion only | 1 (1.9) | 0 (0.0) |  | 0 (0.0) | 0 (0.0) | 0 (0.0) | 0 (0.0) |  | 0 (0.0) |
| **Downstream pathway activation alterations** |  |  |  |  |  |  |  |  |  |
| *PIK3CA* amplification only | 0 (0.0) | 1 (2.9) |  | 0 (0.0) | 0 (0.0) | 0 (0.0) | 0 (0.0) |  | 0 (0.0) |
| *KRAS* mutation only | 0 (0.0) | 0 (0.0) |  | 0 (0.0) | 0 (0.0) | 0 (0.0) | 0 (0.0) |  | 0 (0.0) |
| *BRAF* mutation only | 2 (3.7) | 0 (0.0) |  | 0 (0.0) | 1 (14.3) | 0 (0.0) | 0 (0.0) |  | 0 (0.0) |
| *BRAF* fusion only | 0 (0.0) | 0 (0.0) |  | 0 (0.0) | 1 (14.3) | 0 (0.0) | 0 (0.0) |  | 0 (0.0) |
| *CCND1/CCN E1* amplification only | 0 (0.0) | 0 (0.0) |  | 0 (0.0) | 0 (0.0) | 0 (0.0) | 0 (0.0) |  | 0 (0.0) |
| *CDK4/CDK6* amplification only | 1 (1.9) | 0 (0.) |  | 0 (0.0) | 0 (0.0) | 0 (0.0) | 0 (0.0) |  | 0 (0.0) |
| **Multiple of the above** | 4 (7.4) | 0 (0.0) |  | 0 (0.0) | 1 (16.7) | 4 (22.2) | 0 (0.0) |  | 0 (0.0) |
| **None of the above** | 38 (70.4) | 30 (85.7) |  | 3 (100) | 5 (71.4) | 10 (71.4) | 3 (100) |  | 34 (100) |

1. One patient harbored both *EGFR* Ex19del and *EGFR* L858R mutation.
2. Other *EGFR* mutations include *EGFR* mutations, other than *EGFR* Ex19del, *EGFR* L858R, and *EGFR* T790M mutations.
3. Other mutations include *EGFR* V689L/M, *EGFR* D761N, *EGFR* L62R, *EGFR* R776H, *EGFR* A289T/V, *EGFR* D1012N, *EGFR* E709A, *EGFR* G719A, *EGFR* G724S, *EGFR* L747V, *EGFR* L838V, *EGFR* L861Q, *EGFR* M952I, *EGFR* S306P, *EGFR* V308I, and *EGFR* Q565.

*ALK*, anaplastic lymphoma kinase; *BRAF*, v-raf murine sarcoma viral oncogene homolog B; *CCND1*, cyclin D1; *CCNE1*, cyclin E1; *CDK4*, cyclin-dependent kinase 4; *CDK6*, cyclin-dependent kinase 6; *ERBB2*, v-erb-b2 avian erythroblastic leukemia viral oncogene homolog 2; *EGFR*, epidermal growth factor receptor; Ex19del, exon 19 deletion; FAS, full analysis set; *KRAS*, Kirsten rat sarcoma viral oncogene homolog; *MET*, mesenchymal-epithelial transition; NGS, next-generation sequencing; *PIK3CA*, phosphatidylinositol-4,5-bisphosphate 3-kinase catalytic subunit alpha; *RET*, rearranged during transfection
